# The histone chaperone Nrp1 is required for chromatin stability and nuclear division in *Tetrahymena thermophila*

**DOI:** 10.1186/s13072-021-00409-4

**Published:** 2021-07-23

**Authors:** Yinjie Lian, Huijuan Hao, Jing Xu, Tao Bo, Aihua Liang, Wei Wang

**Affiliations:** 1 Key Laboratory of Chemical Biology and Molecular Engineering of Ministry of Education, Institute of Biotechnology, Shanxi University, 92 Wucheng Rd., Taiyuan, 030006 China; 2 School of Life Science, Shanxi University, Taiyuan, 030006 China

**Keywords:** *Tetrahymena thermophila*, Histone chaperone, Chromatin stability, Nuclear division

## Abstract

Histone chaperones facilitate DNA replication and repair by promoting chromatin assembly, disassembly and histone exchange. Following histones synthesis and nucleosome assembly, the histones undergo posttranslational modification by different enzymes and are deposited onto chromatins by various histone chaperones. In *Tetrahymena thermophila,* histones from macronucleus (MAC) and micronucleus (MIC) have been comprehensively investigated, but the function of histone chaperones remains unclear. Histone chaperone Nrp1 in *Tetrahymena* contains four conserved tetratricopepeptide repeat (TPR) domains and one C-terminal nuclear localization signal. TPR2 is typically interrupted by a large acidic motif. Immunofluorescence staining showed that Nrp1 is located in the MAC and MICs, but disappeared in the apoptotic parental MAC and the degraded MICs during the conjugation stage. Nrp1 was also colocalized with α-tubulin around the spindle structure. *NRP1* knockdown inhibited cellular proliferation and led to the loss of chromosome, abnormal macronuclear amitosis, and disorganized micronuclear mitosis during the vegetative growth stage. During sexual developmental stage, the gametic nuclei failed to be selected and abnormally degraded in *NRP1* knockdown mutants. Affinity purification combined with mass spectrometry analysis indicated that Nrp1 is co-purified with core histones, heat shock proteins, histone chaperones, and DNA damage repair proteins. The physical direct interaction of Nrp1 and Asf1 was also confirmed by pull-down analysis in vitro. The results show that histone chaperone Nrp1 is involved in micronuclear mitosis and macronuclear amitosis in the vegetative growth stage and maintains gametic nuclei formation during the sexual developmental stage. Nrp1 is required for chromatin stability and nuclear division in *Tetrahymena thermophila*.

**Supplementary Information:**

The online version contains supplementary material available at 10.1186/s13072-021-00409-4.

## Introduction

Eukaryotic cells compact genomic DNA into chromatin to fit inside the nucleus. The nucleosome is the basic subunit of chromatin. It contains two copies of core histone and DNA wrapping around the histone octamer with an approximate length of 146 bp [1, 2]. H1 associated with linker DNA links adjacent nucleosomes for high-order chromatin structuring [3]. The structural and functional diversity of nucleosomes is produced by posttranslational modifications of the histones and by histone variants [4]. When the chromatin structure undergoes dynamic changes, histones are not continuously associated with DNA. Newly synthesized histones need to be escorted and transported into the nucleus and targeted to the required location, while old or damaged histones are discarded [5]. Free basic histones are harmful to cells because of disorganized interactions and aggregation. Thus, cellular histones are not present in isolation but are instead complexed with other proteins that neutralize their positive charge [5, 6]. Histone chaperones accompany different histones to interact with DNA and other macromolecules and facilitate nucleosome formation or disassembly [7, 8].

According to binding specificities and sequences and structural similarities, histone chaperones are classified into several families [9, 10]. One family is the nuclear autoantigenic sperm protein (NASP) family, which is also known as the N1/N2 family [11]. In *Saccharomyces cerevisiae*, NASP homolog Hif1 (histone acetyltransferase 1-interacting factor 1) interacts with histone acetyltransferase Hat1 and Hat2 to form the nuclear HAT-B complex, which recognizes the post-translational modifications of the H3 tail and catalyzes the acetylation of the histone H4 tail. Hif1 interacts with the H2A-H2B dimer and H3-H4 tetramer via distinct mechanisms [12–14]. In *Schizosaccharomyces pombe,* NASP homolog Sim3 (start independent of mitosis 3) specifically deposits centromere histone H3 [15, 16]. NASP-1 forms transcriptional repressor complex with histone deacetylase HDA-1 and zinc finger-containing protein Tra4 to repress male-specific genes expression and promote female development in *Caenorhabditis elegans* hermaphrodites [17]. In *Xenopus laevis*, N1/N2 allows the progressive release of histones after fertilization and thus ensures nucleosomal assembly during rapid cell divisions in early development [18, 19]. Reducing mammalian NASP in tissue culture cells results in a defect in cell proliferation. The lack of NASP function in mice causes early embryonic lethality [17, 20]. *Arabidopsis* NASP binds the histone variant CenH3 and affects its abundance at the centromeres [21]. Mammals have two alternatively spliced isoforms of NASP: testis-specific NASP (tNASP) and somatic form of NASP (sNASP). The NASP splice variants are present in most vertebrate species and generate functional diversity in somatic and germline cells. tNASP is found in cancer, embryonic, and germ cells, it is a HSP90 cochaperone for the assembly of the H3-H4 units. sNASP is highly expressed in all dividing cells, it is part of a core complex composed of the HAT1 holoenzyme (composed of the RbAp46 and HAT1 proteins) and H3-H4 [22–25]. The members of NASP family have a plethora of interacting partners, through which they are involved in many different aspects of nuclear metabolism. The potential involvement of NASP in DNA replication, recombination, and repair requires further investigation [26–28].

*Tetrahymena thermophila* is a unicellular ciliated protist that contains structurally and functionally distinct germ-line micronucleus (MIC) and somatic macronucleus (MAC). The polyploid MAC is characterized by transcriptional activity, and the diploid MIC is transcriptionally silent in vegetative growing cells [29]. DNA replication and cell division stop when *Tetrahymena* cells are starved. During the sexual developmental stage, MICs perform meiosis and produce four meiotic products. One of them undergoes a prezygotic mitosis to produce two pronuclei. The exchange and fusion of the pronuclei produce a zygotic nucleus. The zygotic nucleus performs two mitoses and produces four products. The two products at the anterior part form new MACs (NMs), the two products at the posterior part become the new MICs. NMs undergo genome rearrangement and replicate. Finally, the conjugating cells separate and become exconjugants, and one of the MICs is resorbed. The exconjugant resumes proliferation under nutrition conditions [30, 31]. The separation of MAC and MIC is reminiscent of metazoans, where distinct germ cells and somatic cells are maintained [32]. The MAC and the MIC have different histones, histone modification, and chromatin structure. Micronucleus-specific histone H1 (Mlh1) is different from macronuclear H1 (Hho1) and H1 from other organisms [33, 34]. The transcriptionally active MAC contains a histone hvl (H2A variant), and the hvl protein is absent from the MIC, except in the early stages of conjugation [35]. The MIC contains a quantitatively minor H3 that is derived from MAC's H3 by a specific proteolytic cleavage of six amino acid residues [36, 37]. Histone variants and isoforms dynamically regulate chromatin structure and epigenetic signaling to maintain cell homeostasis. These processes require controlled spatial and temporal deposition and the eviction of histones by their dedicated chaperones.

In the present study, we identified a new NASP-related protein 1 (Nrp1) in *T. thermophila*. Nrp1 localized to the MAC and MIC, and disappeared in the apoptotic parental MAC and the degraded MICs. *NRP1* knockdown inhibited cellular proliferation and led to abnormal micronuclear mitosis and macronuclear amitosis during the vegetative growth stage. During conjugation stage, *NRP1* knockdown led to gametic nuclear abnormality and nuclear selection failure. The interaction proteins of Nrp1 were identified by affinity purification combined with the mass spectrometry (AP-MS) analysis of endogenously tagged Nrp1-HA proteins. The physical direct interaction of Nrp1 and Asf1 was also confirmed by pull-down analysis in vitro. These results indicate that Nrp1 is required for chromatin stability and nuclear division in *Tetrahymena*.

## Results

### Characterization of *Tetrahymena* histone chaperone Nrp1

**Fig. 1:**
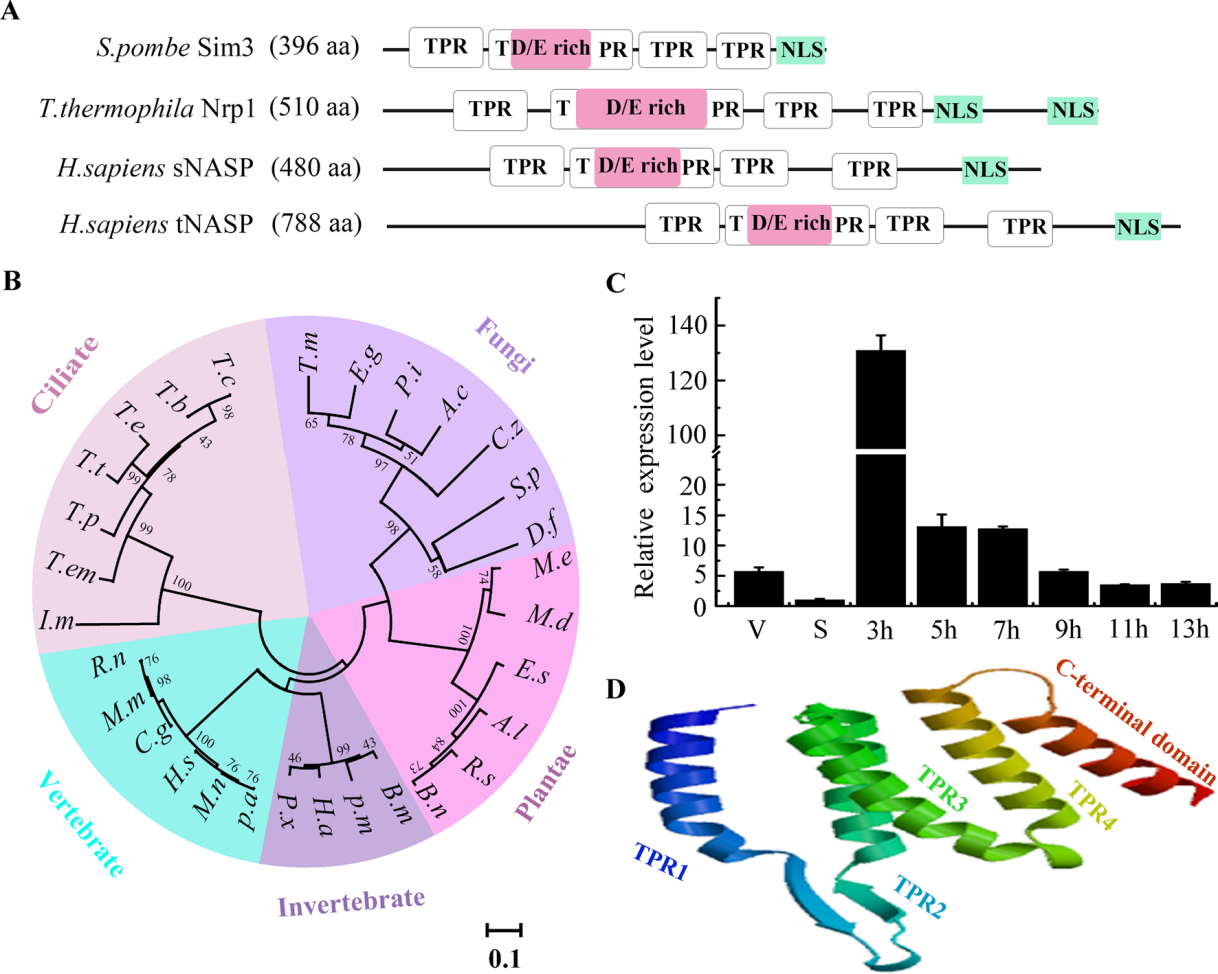
Characterization of *NRP1* from *T. thermophila*. **A** Conserved domain of four different NASP family proteins. TPR indicated tetratricopepeptide repeats; D/E-rich residues indicated rich aspartic and glutamic acid regions. NLS indicated nuclear localization signal peptide. **B** Phylogenetic tree of NASP family proteins from different eukaryotic organisms reconstructed using TPR amino acid sequences. Tree topology and branch lengths correspond to Bayesian inferences. *T.c* (*Tetrahymena canadensis* Nrp1, TSP00244100); *T.b* (*Tetrahymena borealis* Nrp1, TBOREA00197080); *T.e* (*Tetrahymena elliotti* Nrp1, TELLIO00222160); *T.t* (*Tetrahymena thermophila* Nrp1, XP_001030823.3); *T.p *(*Tetrahymena pyriformis* Nrp1, TPYRIF00252750); *T.em* (*Tetrahymena empidokyrea* Nrp1, TEPIDO00181820); *I.m* (*Ichthyophthirius multifiliis* Nrp1, XP_001030823.3); *R.n* (*Rattus norvegicus* NASP, NP_ 001,005,543.1); *M.m* (*Mus musculus* NASP, NP_058057.3); *C.g* (*Cricetulus griseus* NASP, XP 007,609,458.1); *H.s* (*Homo sapiens NASP*, XP_005270945.1); *M.n* (*Macaca nemestrina* NASP, XP_011762308.1); *P.a* (*Pongo abelii* NASP, PNJ21692.1). *P.x* (*Plutella xylostella* NASP, XP_011559258.1); *H.a* (*Helicoverpa armigera* NASP, XP_021184247.1); *P.m* (*Papilio machaon* NASP, XP_014366859.1); *B.m* (*Bombyx mandarina* NASP, XP 028,027,096.1:26–395).* B.n* (*Brassica napus* Sim3, XP_013742238.1); *R.s* (*Raphanus sativus* Sim3, XP 018,450,125.1);* A.l* (*Arabidopsis lyrata subsp* Sim3, XP 020,874,858.1); *E.s *(*Eutrema salsugineum* Sim3, XP 006,411,948.1); *M.d* (*Malus domestica* Sim3, XP 008,377,655.2); *M.e* (*Manihot esculenta* Sim3, XP 021,632,704.1). *D.f* (*Debaryomyces fabry* Sim3, XP_015465497.1); *S.p* (*Schizosaccharomyces pombe* Sim3, NP_595313.1); *C.z* (*Cercospora Zeina* Sim3, XP_015465497.1); *A.c* (*Aspergillus cristatus* Sim3, ODM15912.1); *P.i* (*Penicillium italicum* Sim3, KGO72320.1); *E.g* (*Elaphomyces granulatus* Sim3, OXV08410.1); *T.m* (*Talaromyces marneffei* Sim3, KFX53481.1). **C** Expression profiles of the *NRP1*. The gene fragments were amplified by qRT-PCR at the vegetative stage (V), starvation stage (S), and sexual development (3 h, 5 h, 7 h, 9 h, 11 h, 13 h) stage. **D** The structure of Nrp1 was established with SWISS-MODEL

Histone chaperone NASPs are widely distributed across eukaryotes. They all contain the typical tetratricopepeptide repeat (TPR) domain. The TPR motifs form a helix-turn-helix arrangement and provide a structural scaffold for mediating protein-protein interactions [38]. The *Tetrahymena* genome contains a single NASP homologous gene *NRP1* (TTHERM_01014770)*.* It is 1,700 bp, and the open reading frame is 1533 bp encoding 510 amino acids. Nrp1 contains four tandem TPR domains (TPR1-4) and one C-terminal domain containing the nuclear localization signal (NLS). TPR1, TPR3, and TPR4 consist of α-helices and exhibit similar structures. By contrast, the TPR2 domain is inserted by acidic amino acids patches (Fig. 1A and Additional file 1: Fig. S1A). The identities of amino acid sequences of the four TPR domains from seven different *Tetrahymena* are 94.33%, 73.81%, 84.92%, and 94.16%, respectively. It seems that TPR2 diverges more quickly than the other three TPR domains (Additional file 1: Fig. S1B). The orthologs of Nrp1 are also identified across a wide range of eukaryotic lineages. They are highly conserved in different clusters (Fig. 1B). *NRP1* has low expression during the growth and starvation stage and the expression level is strikingly upregulated during the sexual development stage (Fig. 1C). The *NRP1* expression pattern is consistent with the microarray data from the TFGD (http://tfgd.ihb.ac.cn) (Additional file 1: Fig. S2A). In mammal cells, NASP expression parallels histone expression during the cell cycle, increases during the S phase, declines during G2, and is undetectable in nonmitotic cells [20]. However, the *NRP1* expression profile is different from the histone expression profile during the cell cycle in *Tetrahymena,* because the cell has asynchronous macronuclear and micronuclear cell cycles (Additional file 1: Fig. S2B). The crystal structure of yeast Hif1 shows that Hif1 forms a superhelixed TPR groove domain and a long acid loop covering the rear of the TPR domain [39]. Similarly, Nrp1 mainly contains α-helix, and the TPR2 domain forms a loop region that represents the typical characteristics of SHNi-TPR (Sim3-Hif1-NASP-interrupted TPR) proteins (Fig. 1D).

### Dynamic localization of Nrp1 during vegetative growing stage

**Fig. 2:**
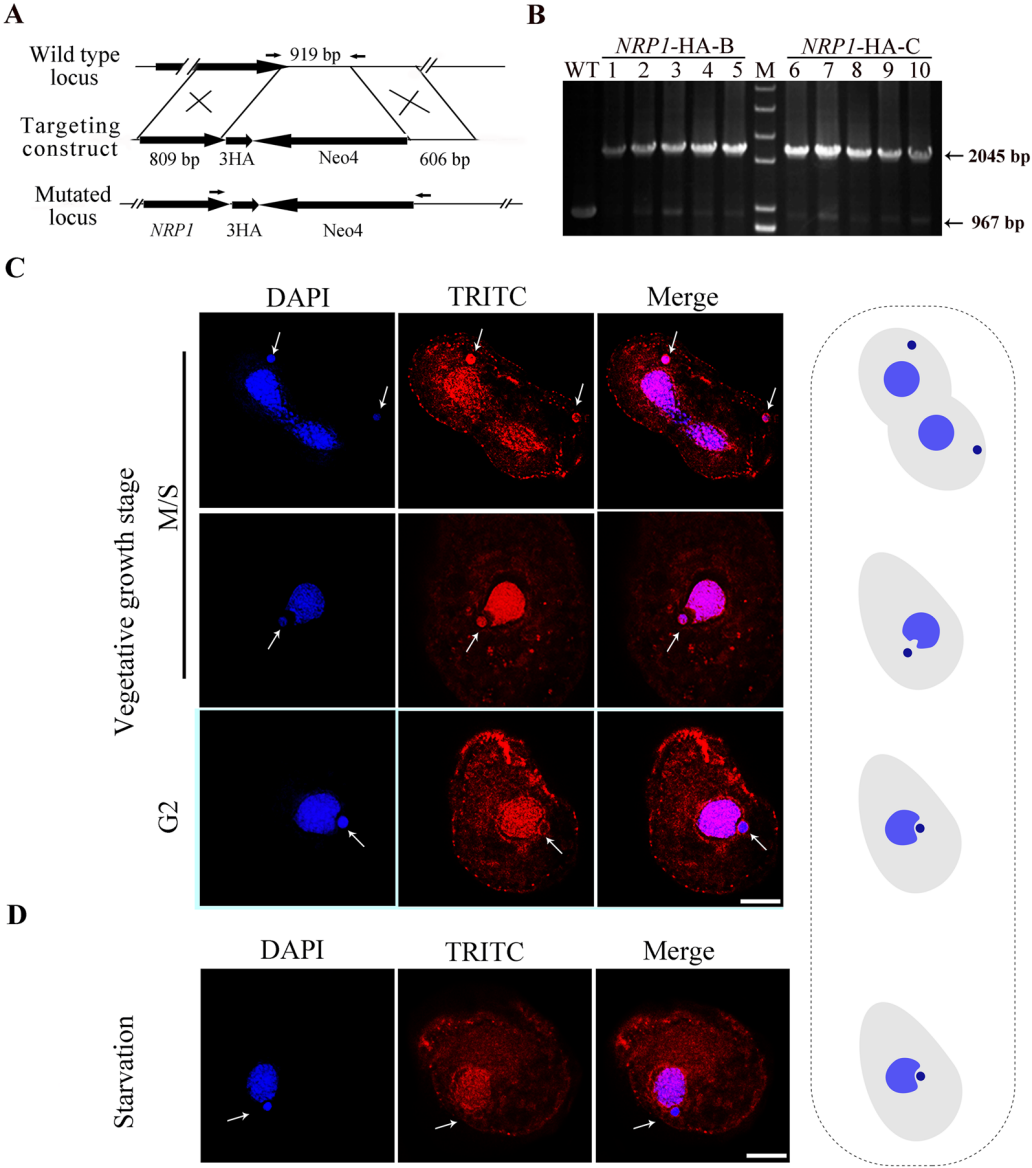
Localization of Nrp1-HA during vegetative growth and starvation stage. **A** Schematic representation for generating recombinant Nrp1-HA mutans in *T. thermophila.* **B** The identification of *NRP1*-HA-B and *NRP1*-HA-C mutants. WT and mutants loci were amplified by PCR. Arrows indicates WT (967 bp) and mutated locus (2045 bp). **C**, **D** Localization of Nrp1-HA during vegetative growth stage and starvation stage. Nrp1-HA cells collected at the vegetative growth stage and starvation stage. The arrows indicated MICs; the right panel is a diagram of the cell development model. Scale bar, 10 µm

To explore localization of Nrp1, a sequence encoding three hemagglutinin (HA) epitopes was inserted into the 3′ end of the Nrp1 open reading frame (Fig. 2A). The p*NRP1*-3HA-Neo4 was constructed and introduced into different mating type cells, CU428 and B2086. The endogenous *NRP1* gene in the MACs was targeted with the HA tag at the C-terminal (Fig. 2B). During asexual reproduction, the MIC divided mitotically, while the MAC divided amitotically (Fig. 2C). Unlike the classical G1-S-G2-M cell division cycle, the MAC performs amitosis-G1-S-G2 and the MIC performs S-G2-M pattern in *Tetrahymena*. The micronuclear M phase and next S phase are compressed into a common interval devoid of an apparent G1 interval [40, 41]. Nrp1 signals were strong in the MIC and MAC in the M/S phases. However, the signal was only discovered around the periphery of the MIC at the G2 phase of the log-phase growing cells or starved cells, while untagged wild-type cells have no specific signal (Fig. 2D, Additional file 1: Fig. S3A). Nrp1-HA expression during vegetative growing stage was confirmed by Western blotting (Additional file 1: Fig. S3B). The periodic appearance of the Nrp1 signal in the MIC was consistent with the NASP expression change in metazoan organisms. The results indicated that Nrp1 could be involved in the micronuclear and macronuclear dynamic organization in the vegetative growth stage.

### Localization of Nrp1 during sexual development stage

**Fig. 3:**
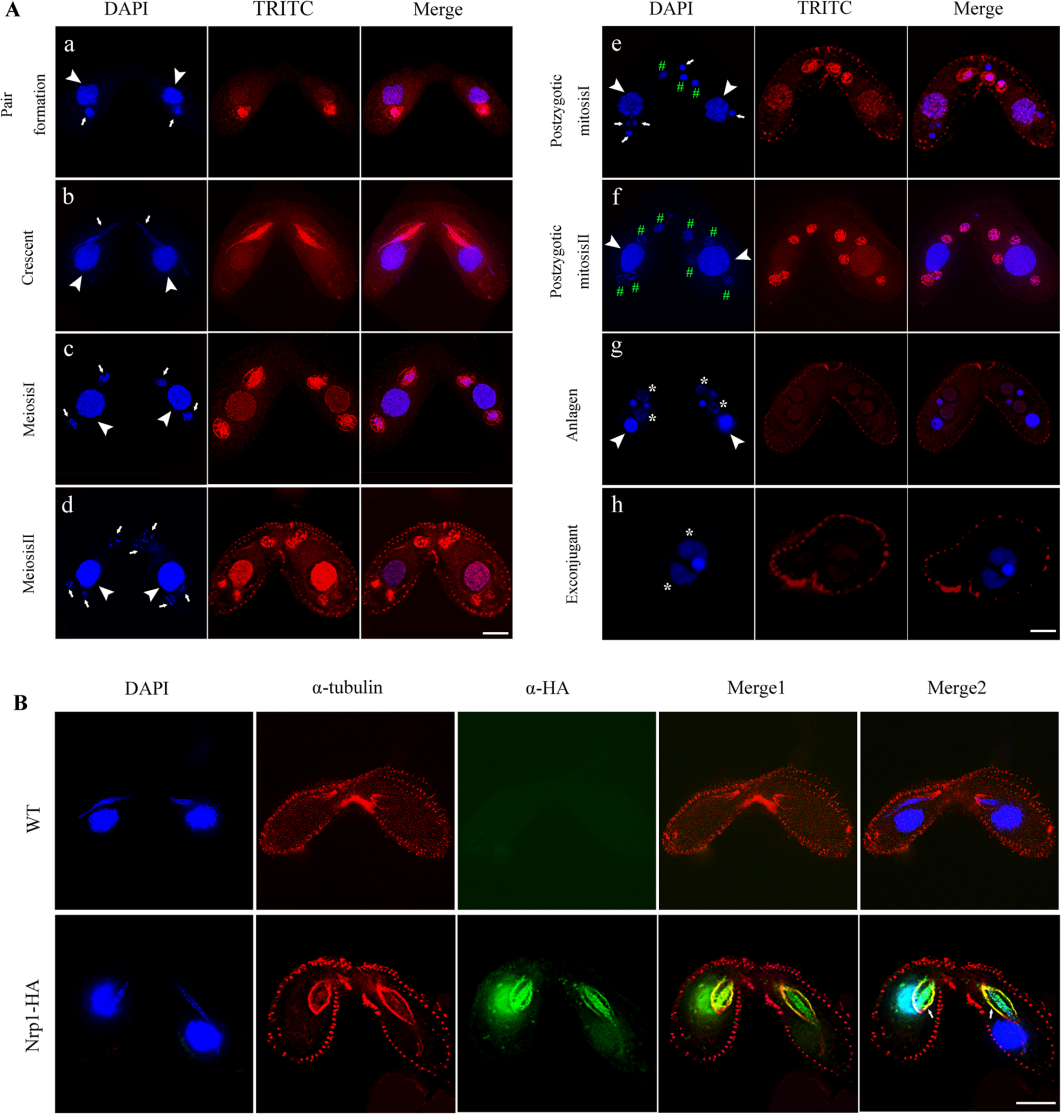
Localization of Nrp1-HA during conjugation stage. **A** Immunofluorescence staining of Nrp1-HA during conjugation stage. DNA was stained with DAPI. a, pair formation; b, crescent; c, meiosis I; d, meiosis II; e, postzygotic mitosis I; f, postzygotic mitosis II; g, anlagen stgage; h, exconjugant stage. **B** Co-localizaiton of Nrp1 and α-tubulin during early conjugation stage. The experiments were repeated three times. White arrowheads indicate parental MACs, arrows indicate MICs, stars indicate anlagen, and pounds indicate postzygotic nucleus. Scale bar, 10 µm

NASP is initially described in rabbits as a highly autoimmunogenic testis and sperm-specific protein and localized in the nuclear area of primary spermatocytes. During the subsequent meiotic divisions, NASP is partitioned into the cytoplasm and then reassociates with the reforming nucleus [22, 42]. During the sexual developmental stage, the Nrp1 signal in the MAC was weak during premeiosis (Fig. 3Aa, b), then the signal gradually increased during meiosis (Fig. 3Ac, d). During postzygotic mitosis, the signal is weakened (Fig. 3Ae, f) and disappeared during anlagen stage (Fig. 3Ag, h). In contrast, Nrp1 strongly localized in the meiotic MICs, mitotic pronuclei, and mitotic zygotes throughout early conjugation stage (Fig. 3Aa, f). The signal disappeared from the degraded MICs and parental MACs during the late conjugation stage (Fig. 3Ae, f). Furthermore, Nrp1 signal was observed in the new developing MACs and new MICs, which were replicated into 32C and 4C, respectively (Fig. 3Ag, Additional file 1: Fig. S3C, S8). In the exconjugant stage, the Nrp1 signal decreased in the new MACs and new MICs (Fig. 3Ah, Additional file 1: Fig. S3C, S8). Interestingly, the Nrp1 signal covered more than the 4′,6-diamidino-2-phenylindole dihydrochloride (DAPI)-stained areas in the MICs (Fig. 3Ab–e). These localization signals are similar to spindle structure. Therefore, we did the colocalization of Nrp1 and α-tubulin. The results showed that Nrp1 and α-tubulin forms colocalization around the spindle apparatus, thereby implying that Nrp1 may be involved in spindle function (Fig. 3B). These results indicated that Nrp1 is involved in the macronuclear transcription, micronuclear DNA replication, and micronuclear mitotic division during the sexual development stage.

### *NRP1* knockdown affects DNA replication and chromatin stability

**Fig. 4:**
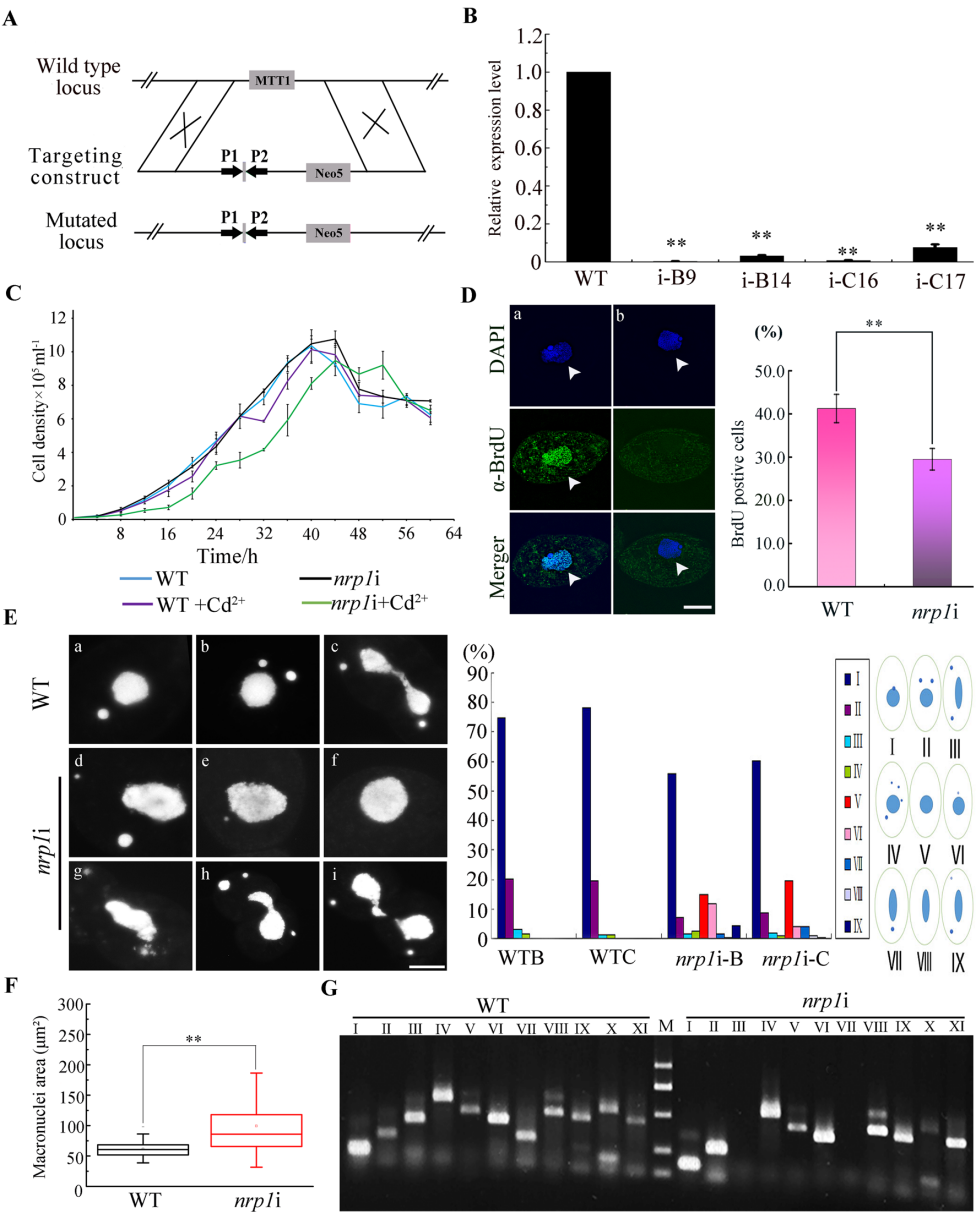
Micronuclear mitosis and macronuclear amitosis *in nrp1*i mutants. **A** Schematic representation for generating *NRP1* knockdown mutants. **B** Identification of *NRP1* interference efficiency. Total RNA was isolated from the vegetative growing WT and *nrp1*i cells. The cells were induced with 0.5 µg/mL Cd^2+^ for 96 h. The relative expression level of *NRP1* was identified by qRT-PCR. **C** Proliferation of *nrp1*i mutant and WT. **D** Replicated DNA was labeled with BrdU. Percentage of BrdU-positive (n = 300) in WT cells and *nrp1*i mutants, respectively. Scale bar, 10 µm. The arrowhead indicates the MAC. **E** Representative of the division of MIC and MAC. Lost MIC and abnormally divided MAC was showed in the *nrp1*i mutants. **F** Statistical analysis of MAC size in WT cells and *nrp1*i mutants (n = 100). **G** MIC-specific sequences were amplified by PCR with 10 sets of primers. Primers II to XI were designed for five different chromosomes in MIC, respectively. The loci on chromosomes IV and V were lost in *nrp1*i mutants. JMJ1 was used as the internal control

To explore the function of Nrp1, we constructed *NRP1* knockout plasmid and transformed it into *Tetrahymena* cells (Additional file 1: Fig. S4A). The *NRP1* knockout transformants were selected and screened under paromomycin resistance. *NRP1* alleles were only partially replaced by the Neo4 cassette. We failed to obtain *NRP1* completely knockout strains, because *NRP1* was required for vegetative growth (Additional file 1: Fig. S4B, C). Furthermore, *NRP1* knockdown mutants were created by the conditional RNA interference (RNAi) method. Double-strand RNA was induced under the MTT1 promoter by Cd^2+^ induction, and target gene transcripts were disrupted by small RNA [43, 44]. The pNRP1hpNeo plasmid was constructed and transformed into *Tetrahymena* cells (Fig. 4A). Different mating-type *nrp1*i mutants were obtained. The *nrp1*i cells were induced by Cd^2+^ during the vegetative growth stage. The expression levels of mutant B9 and mutant C16 were down-regulated by 50-fold (Fig. 4B). The proliferation of the *nrp1*i mutants was inhibited (Fig. 4C). Furthermore, single *nrp1*i cell was cloned into SPP medium drops, and their proliferation was assessed (n = 200). For WT cells, each single cell survived and proliferated. By contrast, 19.5% *nrp1*i cells died, and 36.5% *nrp1*i cells failed to proliferate (Additional file 1: Fig. S5). The results indicated that *NRP1* is essential for cellular proliferation in *Tetrahymena*.

To determine whether Nrp1 directly affects DNA replication, we labeled WT and *nrp1*i cells for 2.5 h with BrdU. The BrdU incorporation number in *nrp1*i cells significantly decreased (P < 0.01) than that in WT cells (Fig. 4D). 38.4% *nrp1*i-B9 and 27.7% *nrp1*i-C16 cells showed unequal micronuclear mitosis and abnormal macronuclear amitosis (Fig. 4Ed–i). Furthermore, 14.8% *nrp1*i-B9 and 19.6% *nrp1*i-C16 cells lost MICs during cell proliferation (Fig. 4Ef). The abnormal division of MAC also occurred and formed large chromatin extrusion (Fig. 4Eh). In addition, MAC size increased in the *nrp1*i cells (Fig. 4F). During sexual development, the germline genome of the *Tetrahymena* undergoes programmed chromosome breakage and massive DNA elimination to generate the somatic genome [45, 46]. The MAC chromosomes are generated by cleavage at chromosome breakage sequences (CBS) that are consecutively spaced along the MIC chromosome. Specific primers can be used to amplify MIC-specific sequences [33]. With the PCR assay, the integrity of five chromosomes was analyzed in *nrp1*i cells. The left arm of the V chromosome and the right arm of the IV chromosome were lost (Fig. 4G). These results demonstrated the *NRP1* knockdown affects DNA replication, disturbs chromatin stability, and inhibits *Tetrahymena* proliferation.

### *NRP1* knockdown affects gamete formation during sexual developmental stage

**Fig. 5:**
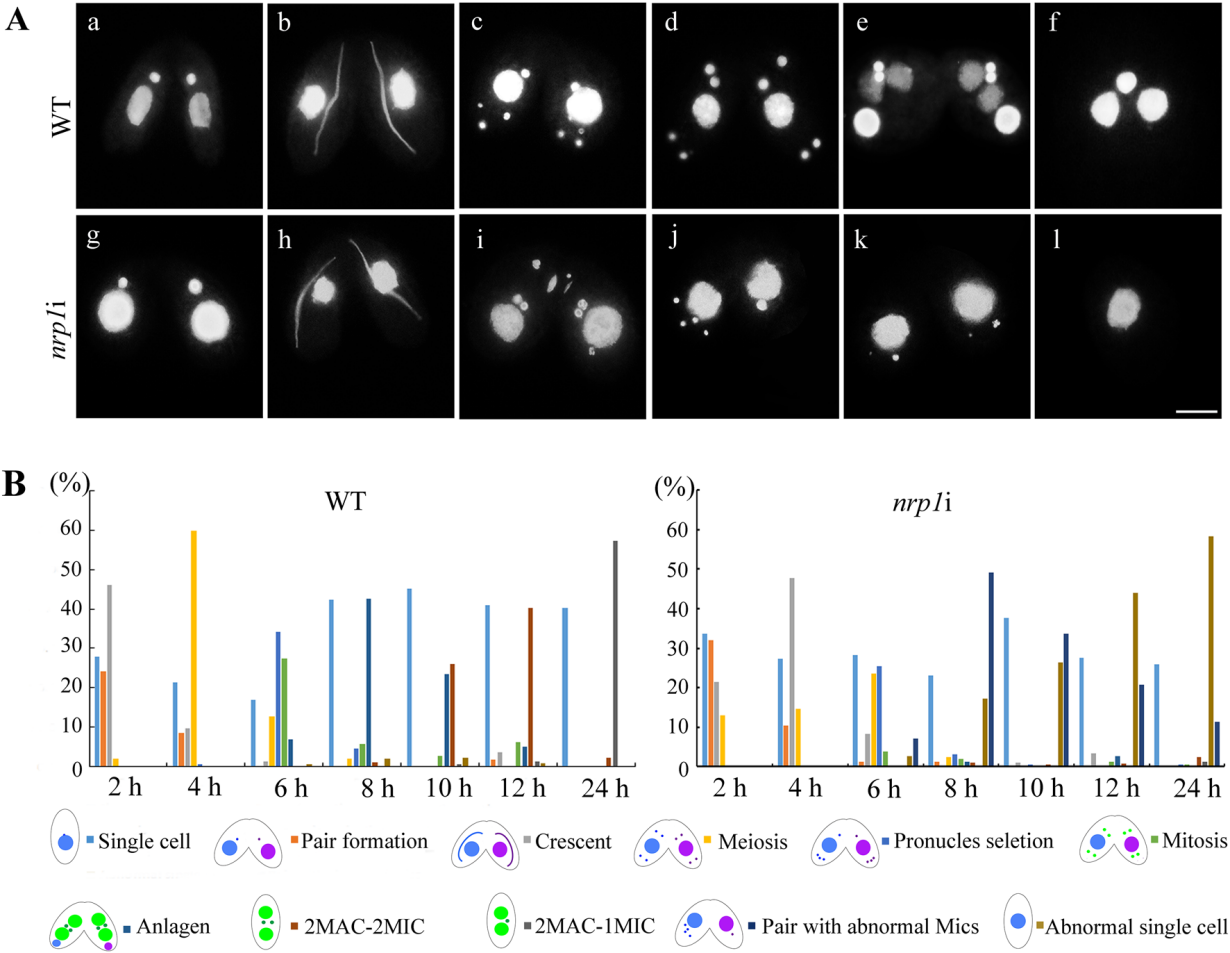
Sexual developmental progression of *nrp1*i mutants. **A** Gametic nuclei lost during the conjugation stage in *nrp1*i mutants. a and j, pair formation; b and h, crescent; c and i, nuclear selection; d, postzygotic mitosis; e, anlagen stage; f, exconjugant. j, k, and l indicated abnormal developing cells. DNA was stained with DAPI. Scale bar, 10 µm. **B** Percentage of different developmental stages during the conjugation stage in WT cells and *nrp1*i mutant cells (n > 300)

Five nuclear divisions, namely, three prezygotic and two postzygotic divisions, occur in each cell during *Tetrahymena* sexual development [32]. To explore the function of Nrp1 during the sexual development stage, different mating type *nrp1*i cells were induced by Cd^2+^ for 24 h during the starvation stage. The starved cells were mixed and then initiated sexual development progress. During the early stages of conjugation, *nrp1*i mutants formed normal mating pairs as the WT cells (Fig. 5Aa–b, Ag–h). With conjugation development, the mating *nrp1*i mutants displayed abnormal meiosis phenotypes, including the unequal segregation of chromosomes (Fig. 5Ai). More than 68.5% of the cells completed pronuclear selection in the WT cells (Fig. 5Ac, B), while only 32.1% cells passed the stage in the *nrp1i* cells (Fig. 5B). In the subsequent developmental process, most of the *nrp1*i mutants failed to complete zygotic mitosis (Fig. 5Aj, k) and finally degraded the abnormal MICs (Fig. 5Al). Only 3% of the *nrp1*i mutants developed into the anlagen stage. Finally, 57.4% WT and 1.1% *nrp1*i mutants completed the sexual development process (Fig. 5B).

**Fig. 6:**
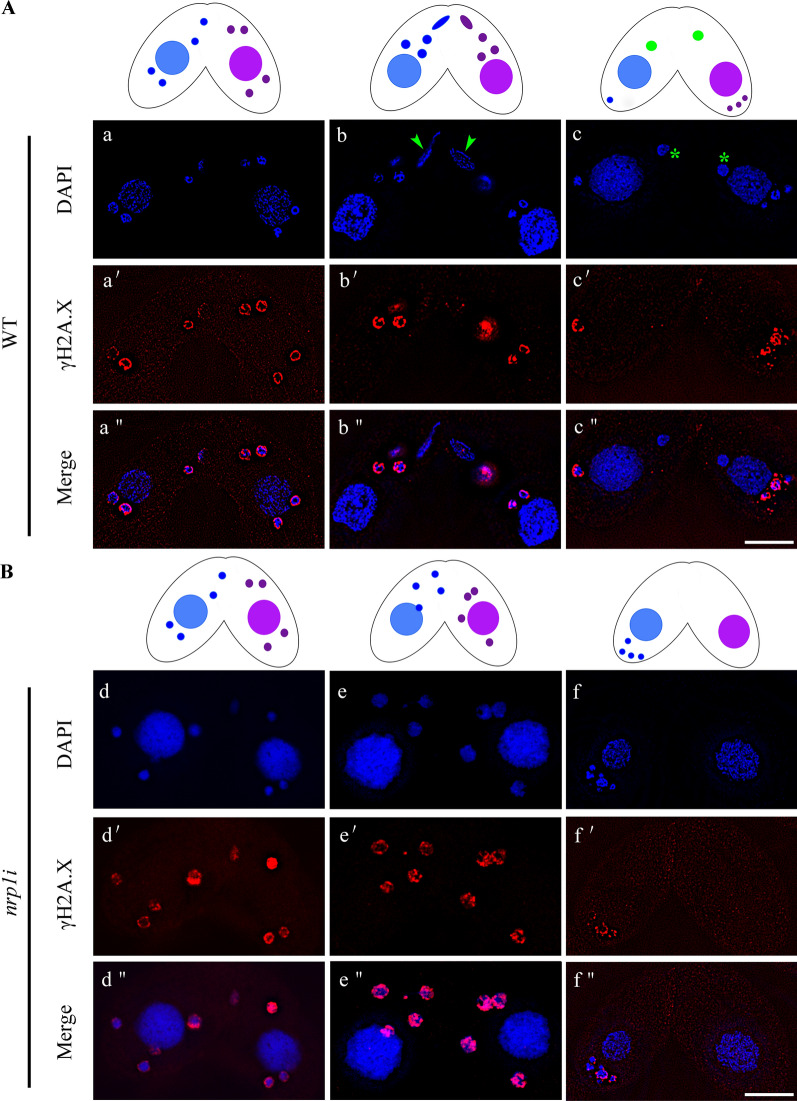
Immunofluorescence staining of γ-H2A.X. Immunofluorescence staining of γ-H2A.X after completion of micronuclear meiosis. Post-meiotic DSBs triggers H2A.X phosphorylation. The unselected pronuclei retaining γH2A.X are eventually eliminated. **A** γ-H2A.X signal occurred in the meiotic MICs and disappeared in the selected pronuclei in WT cells. a/a′/a′′, post-meiotic γ-H2A.X formation; b/b′/b′′, pronuclear selection; c/c′/c′′, zygotic formation. **B** γ-H2A.X occurred and maintained in the meiotic MICs and disappeared with the MIC degradation in the *nrp1*i mutants. d/d′/d′′, post-meiotic γ-H2A.X formation; e/e′/e′′, pronuclear selection; f/f′/f′′, degradation of micronuclei. The arrows indicate the selected pronuclei. The stars indicate zygotes. Scale bar, 10 µm

*Tetrahymena* undergoes a spermiogenesis-like post-meiotic stage, in which transient DSBs form and perform a dynamic change in the chromatin structure of gametic nuclei prior to fertilization [47]. To further test whether the post-meiotic DSB repair was affected, we stained the *nrp1*i and WT cells with antibodies against γ-H2A.X during early conjugation stage. The γ-H2A.X signal occurred primarily in the crescent MIC and disappeared in the later stages after DSBs were repaired (Additional file 1: Fig. S6). In one of the four pronuclei, γ-H2A.X fluorescence disappeared, but the signal persisted in the other three degraded pronuclei in mating WT cells (Fig. 6A). However, the γ-H2A.X signal persisted in the four post-meiotic nuclei in the *nrp1*i cells until they were all degraded (Fig. 6B). The selected pronuclei failed to repair in mating *nrp1*i mutants. These results showed that Nrp1 is required for micronuclear chromatin repair during meiosis in *Tetrahymena*.

### Identification of Nrp1 interaction network

**Fig. 7:**
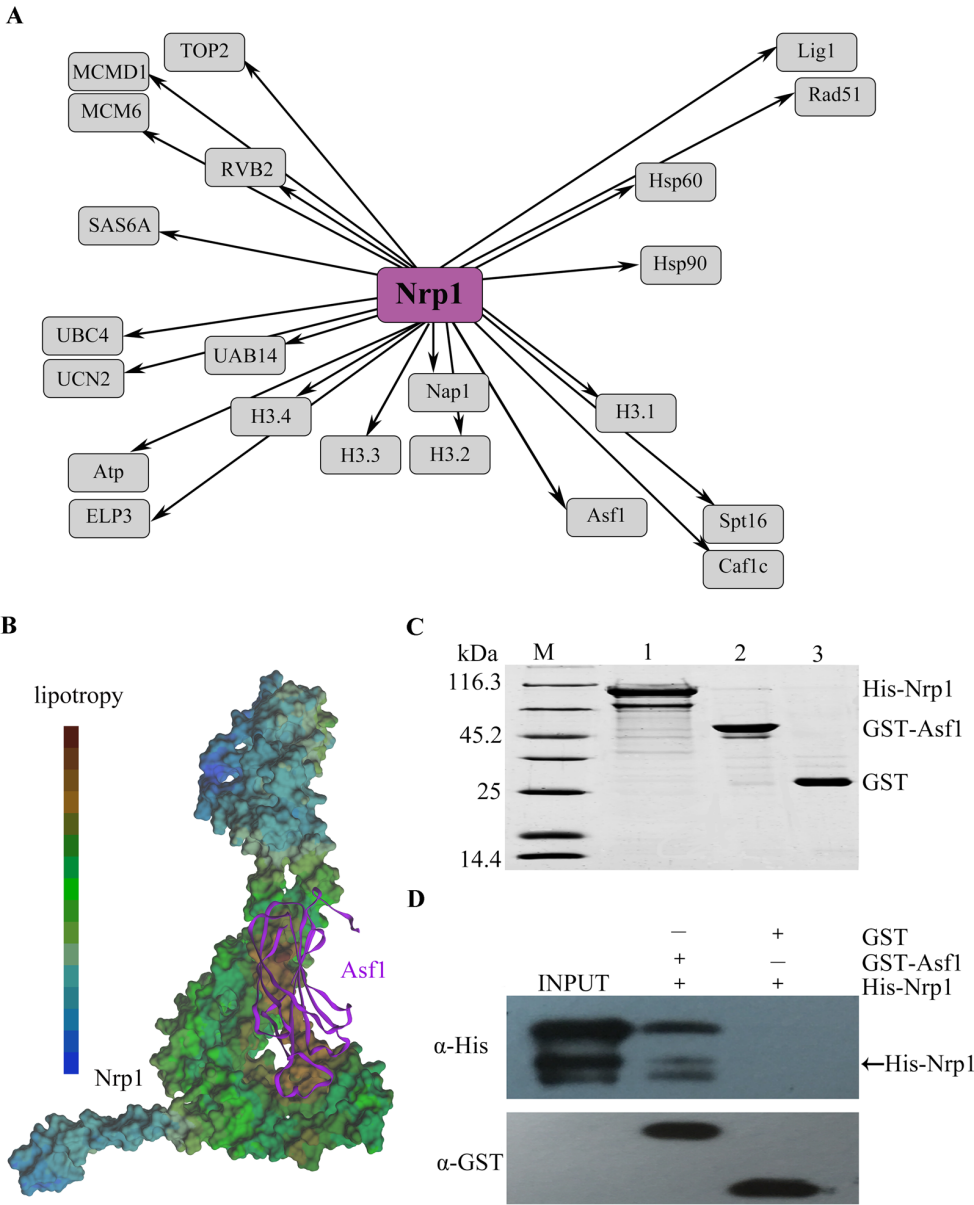
Interaction network of Nrp1-HA and direct interaction of Nrp1 and Asf1. **A** Interaction network view of Nrp1-HA. The bait node is shown in purple. The distance and location of the prey node are shown according to their frequency and putative functions. **B** The structure of the Nrp1-Asf1 complex was predicted by Z-DOCK. The predicted structures and lipotropic property were viewed and rendered using PyMOL. **C** 12.5% SDS-PAGE analysis of His-Nrp1 and GST-Asf1. M: protein molecular weight markers; lane 1: purified His-Nrp1; lane 2: purified GST-Asf1; lane 3: purified GST. **D** Pull down assay confirms the interaction between Nrp1 and Asf1. Recombinant His-Nrp1 and GST-Asf1/GST were incubated with glutathione sepharose resin. Unbound proteins were washed off. The protein complexes were eluted and immunoblotted with anti-GST and anti-His antibodies, respectively

Human NASP interacts with H3-H4 in various multichaperone complexes, including combinations of other H3-H4 chaperones, such as CAF-1, HIRA, and Asf1 [48]. To identify the Nrp1 signaling pathway, we identified the interaction proteins of Nrp1 through the AP-MS analysis of the Nrp1-HA proteins. The expression of *NRP1* was upregulated during early conjugating stage. To obtain abundant interacting proteins, conjugating cells were collected. Nrp1-HA expression was confirmed by Western blotting (Additional file 1: Fig. S7). Nrp1 was copurified with 22 significant interacting partners, including different histones (H3.1, H3.2, H3.3, H3.4), heat shock family proteins (Hsp60 and Hsp90), histone chaperones (Asf1, Caf1c, Spt16, Nap1), DNA replication-related proteins (TOP2, MCMD1, MCM6 and RVB2), and DNA damage repair proteins (Rad51 and Lig1). In *Tetrahymena*, H3.1 and H3.2 are deposited onto the chromatin when DNA is synthesized during DNA replication and meiotic recombination. By contrast, H3.3 and H3.4 deposition is DNA replication-coupled and DNA replication-independent [36]. Thus, Nrp1 is probably involved in replication-dependent and replication-independent chromatin assembly. In HeLa cells, Hsp70 and Hsp90 fold newly synthesized histone H3.1 and histone H4. NASP is a HSP90 cochaperone for the assembly of the H3.1-H4 units [24]. Hsc70 and Hsp90β promote the chaperone-mediated autophagy (CMA) and the depletion of Hsc70 or Hsp90β leads to a striking increase in the levels of soluble H3-H4 [49]. The H3-H4 dynamic balance is maintained between degradation by CMA and protection by NASP in HeLa S3 cells [50]. Herein, we also found that Nrp1 interacts with two heat shock family proteins. sNASP associates with RbAp48 and Hat1 in HeLa S3 lineage and interacts with Hat1p/Hat2p in *S. cerevisiae*. This complex promotes the Hat1-mediated acetylation of Lys5 and Lys12 on the H4 N-terminal tail [12, 24, 51]. DNA replication-related proteins and DNA damage repair-related factors are also detected in the complexes of Nrp1. Rad51 is involved in DNA break repair in the meiosis of *Tetrahymena* [52]. NASP is phosphorylated after DNA damage and appears during DSB repair in 293 T cells [28]. The phosphoproteomic analysis of *Tetrahymena* and AP-MS data also shows that Nrp1 is modified by phosphorylation (http://tfgd.ihb.ac.cn). We also identified ubiquitin degradation pathway-associated proteins UAB14, UBC4, and UCN2. NASP is regulated by the ubiquitin-proteasome pathway in HeLa cells [53]. In addition, the spindle assembled proteins SAS6A, acetyltransferase Atp, and ELP3 were also found in the complexes (Fig. 7A; Additional file 1: Table S2). Although sNASP was initially described as a linker histone chaperone, histone H1 was not detected in the cytoplasmic core complex of sNASP in the HEK293F cells and in *Arabidopsis* NASP complex [24]. We also failed to identify the macronuclear H1 Hho1 and micronuclear H1 Mlh1 in the Nrp1 complex.

Asf1 co-purifies with NASP in human cells. Similar to their human counterparts, yeast Asf1 interacts with Hif1, and the interaction is likely mediated by histones H3/H4 [13]. The AP-MS data of the co-purification with Asf1-FZZ from vegetatively growing cells and conjugation cells revealed the interaction of Asf1 and Nrp1 in *T. thermophila* [54]. The directed interactions of Nrp1 and Asf1 were investigated by Z-DOCK software. Asf1 was combined with the groove of Nrp1 (Fig. 7B). To further confirm the physical interaction of Asf1 and Nrp1, *NRP1* and *ASF1* were synthesized and expressed in *E. coli* BL21. His-Nrp1 and GST-Asf1 were expressed and purified by affinity chromatography (Fig. 7C). The direct physical interaction between Nrp1 and Asf1 was confirmed by pull-down analysis in vitro (Fig. 7D).

## Discussion

NASP protein is widely distributed throughout eukaryotes, and it is a generalized histone chaperone that most likely existed in the last eukaryotic common ancestor [11]. Mammalian NASP and *X. laevis* N1/N2 share 50% identity [42, 55]. Arabidopsis NASP shares 25% and 27% sequence identity with its homologs in fission yeast and mouse, respectively [56]. *Tetrahymena* Nrp1 shares 38% identity with its homolog in *Paramecium* (Additional file 1: Fig. S1A). Phylogenetic analysis revealed that the NASP homologs of ciliates were clustered and independent from the metazoan clade. However, they all contained characteristic TPR domains. TPR2 was interrupted by acidic D/E-rich residues. The acidic patches of sNASP were essential for linker histone binding, and TPR4 played a critical role in the interactions with histone H3-H4 complex [57]. TPR1 and TPR4 exhibited higher divergence than TPR2 and TPR3 [11]. However, we found that TPR2 diverged more quickly than the other three TPR domains in seven different *Tetrahymena* species (Additional file 1: Fig. S2A). The NASP splice variants are present in most vertebrate species and exhibit different functions [27]. Furthermore, some organisms undergo gene duplication events and contain NASP paralogs. The NASP paralogs exhibit a polyphyletic origin [11]. However, *Tetrahymena* contains a single *NRP1* gene and functions in MAC and MIC, which could have evolutionarily functional diversity.

### Nrp1 is closely related to DNA replication and nuclear division

NASP expression is tightly cell cycle regulated in mouse 3T3 cells and HeLa cells [22]. The histones are transported to the various subcellular compartments by chaperons. In HeLa cells, NASP localizes in the nucleus and cytoplasm [58]. In mammalian testis, NASP is localized in the nuclear area of primary spermatocytes. During the meiotic divisions, NASP is partitioned into the cytoplasm and then reassociates with the reforming nucleus [42, 59]. Hif1 mainly localizes in the nucleus in *S. cerevisiae* [14]. *Arabidopsis* NASP is a ubiquitous soluble nuclear protein that is not tightly bound to chromatin [56]. In *Tetrahymena,* the localization pattern of Nrp1 changes periodically. During the vegetative growth stage, the MIC divides mitotically, and the MAC divides amitotically. Nrp1 located in the MIC during the S and M phases. The signal was not strongly detected in the G2 phases. When *Tetrahymena* cells are starved for 24 h, DNA replication and cell division stop, with the MAC in the G1 phase and MIC in the G2 phase [60]. Nrp1 only located in the MAC, and disappeared in the MIC. Obviously, Nrp1 is involved in DNA replication-independent nucleosome assembly in the MAC in the starvation stage. In sexual reproduction, the MIC DNA content does not change during meiosis, DNA synthesis is associated with DNA repair following meiotic recombination between maternal and paternal chromosomes [61]. Nrp1 preferentially located in the elongated crescent MIC during early conjugation stage. The results implied that Nrp1 is involved in the meiotic recombination and programmed DNA repair. Following MIC meiosis, one of the four meiotic products underwent a prezygotic mitosis to produce two pronuclei [62]. Nrp1 strongly localized in the selected pronuclei. However, the signal disappeared from the other degraded MICs. The zygotic nucleus produced four products through two postzygotic mitoses [29]. The Nrp1 signal improved during the zygote mitosis stage. The results strongly showed that Nrp1 is involved in the DNA replication progress. However, the signal was weak in the new MAC, which is replication active. We speculate that parental Nrp1 degraded, new Nrp1 expressed by new developing MACs exert the function in late conjugation stage. Indeed, overexpressed HA-Nrp1 strongly localized in the new developing MAC (Additional file 1: Fig. S8C). We also found the localization signal of Nrp1 was similar to that of spindle apparatus and colocalized with α-tubulin in these regions. The results imply that Nrp1 is involved in the spindle function and promotes nuclear division. Post-meiotic DSBs are necessary to support chromatin remodeling during spermiogenesis. *Tetrahymena* undergoes a spermiogenesis-like post-meiotic stage [47, 61]. The DNA damage occurs in all pronuclei of *Tetrahymena* during post-meiotic stage, but the selected pronucleus specifically undergoes DNA repair and the remaining unselected pronuclei degrade [63]. Loss of Nrp1 inhibits repair of the pronucleus and leads to the degradation of all the pronuclei. Top2β and Spo11 produce transient DSBs in the haploid chromosomes to support chromatin remodeling [47]. Nrp1 is involved in the chromatin remodeling and DSBs repair progress to produce mature gametes.

### Nrp1 interacts with core histones and chromatin assembly factors

tNASP knockdown effectively inhibits the proliferation and causes G1 phase arrest through the ERK/MAPK signaling pathway in renal cell carcinoma cells. tNASP is also critical for the survival of prostate cancer cells [22, 64]. Without sufficient NASP, HeLa and U2OS cells are unable to replicate their DNA and progress through the cell cycle [20]. NASP levels in liver tumors are generally higher than those in normal liver tissues, and NASP down-regulation inhibits liver cancer cells from forming tumors [65]. In *S. pombe* cells, Sim3 is required for central core silencing and normal chromosome segregation [15, 16]. In *Arabidopsis* cells, NASP binds with the histone variant CenH3 and affects its abundance at the centromeres. Reduced NASP expression negatively affects CenH3 deposition at the centromeres [21]. In *Tetrahymena*, MIC is diploid and consists of five pairs of chromosomes, whereas the MAC is polyploidy and consists of 181 chromosomes [32]. During vegetative growth, the MIC divides through mitosis, whereas the MAC divides through amitotic process [66]. The MAC chromosome segregation proceeds independent of any centromere function. The centromeric histone Cna1p specifically localizes to centromeres in the MIC [67]. Cna1 is essential for normal micronuclear DNA segregation during mitosis [68]. In this work, *NRP1* knockdown affected DNA replication, disturbed micronuclear mitosis and macronuclear amitosis, and inhibited *Tetrahymena* proliferation in the vegetative growth stage. Nrp1 knockdown not only affected core histone H3 deposition in the MAC and MIC, but also could disturb Cna1p deposition in the MIC, because Nrp1 is a unique NASP homolog in *Tetrahymena*. Furthermore, Nrp1 localized to micronuclear chromosomes and the spindle structure (Fig. 3B). Interestingly, Cna1p is also localized on the meiotic spindle akin to chromosomal passenger proteins [67].

In *S. cerevisiae,* Hif1 interacts with H3/H4, histone acetyltransferase complex, chromatin assembly proteins, DNA replication-associated proteins, DNA damage response proteins, transcription regulation-related proteins, and RNA polymerase II transcriptional pre-initiation complex assembly [13]. Hif1 buffers displaced histones during transcription and makes them available for immediate chromatin reassembly. *Arabidopsis* NASP binds with heat shock protein HSC70-1, acetyltransferase, histone variant CenH3, histone H3.1, histone H3.3, subunit of Caf1, and Nap1 [56]. In mammals, sNASP promotes chromatin assembly in the presence of core histones and yeast cytosolic extract. sNASP is not only a histone-binding protein but also a chromatin assembly factor [14, 24]. In *Tetrahymena*, we also found that Nrp1 interacted with different core histones, heat shock family proteins, chromatin assembly proteins, DNA replication-associated proteins, DNA damage repair-related proteins and spindle assembled proteins. However, we failed to identify the centromeric histone Cna1p, the macronuclear H1(Hho1), and micronuclear H1(Mlh1) in the Nrp1 complex. Similarly, Nrp1 is also devoid in the Hho1 and Mlh1 complex [69]. Perhaps they have weak interaction, because they exhibit some similar localization pattern. Nrp1 facilitates the assembly of a multiprotein complexes and leads to the diversity of cellular functions. However, peptide count is low and more experimentation is required to fully characterize the Nrp1 interaction network. The affinity and specificity of different target protein binding is still deserved to be further investigated in future. Taken together, Nrp1 is necessary for the macronuclear amitosis, micronuclear mitosis, and meiosis throughout different development stage. It forms different complexes, which is involved in chromatin replication, assembly, and repair progress in *Tetrahymena*.

## Materials and methods

### Strains and culture

Wild-type *T. thermophila* cells B2086 (mating type II), CU428 (mating type VII), and CU427 (mating type VI) were obtained from the National *Tetrahymen*a Stock Center (http://tetrahymena.vet.cornell.edu/). Tetrahymena cells were cultured in Super Proteose Peptone (SPP) medium at 30 °C [70]. The cells were starved in the 10 mM Tris, pH7.4 at 30 °C without shaking for 18–24 h [71]. Mating was induced by mixing equal numbers (~ 2 × 10^5^ cells/mL) of starved cells from different mating types.

### Identification of *NRP1*

*NRP1*(TTHERM_01014770) sequences were obtained from the *Tetrahymena* Genome Database (http://www.ciliate.org). Alignments of amino acid sequences were performed with DNAMAN. Protein clustering analyses were accomplished using NSAP sequences from 34 different eukaryotic lineages by employing the neighbor-joining (NJ) method with 1,000 bootstraps in MEGA 5.1. Structural and functional domains were identified on the basis of the information from the Conserved Domain Database (http://www.ncbi.nlm.nih.gov/Structure/cdd/cddsrv.cgi). Tertiary structure prediction was carried out using the SWISS-MODEL server (https://swissmodel.expasy.org/interactive/).

### Protein HA tagging

The p*NRP1*-HA-Neo4 plasmid was created as previously described [72]. Briefly, the 809 bp C-terminal fragment and 606 bp flanking sequence of *NRP1* were amplified from genomic DNA by PCR using primers the *NRP1*-HA-F1/*NRP1*-HA-R1 and *NRP1*-HA-F2/*NRP1*-HA-R2, respectively (All the primers used in the study are listed in Additional file 1: Table S1). The PCR products were cloned into the pGM-19 T vector and confirmed by sequencing. Then C-terminal fragment digested with *Sac* I and *Not* I, and the flanking sequence digested with *Xho* I and *Kpn* I were ligated with the pHA-Neo4 vector digested with the same enzyme. The plasmid p*NRP1*-HA-Neo4 was introduced into the starved *Tetrahymena* cells using the biolistic particle transformation system GJ-1000 (SCIENTZ, China). The transformed cells were selected under paromomycin and identified by PCR using primers J-*NRP1*-HA-F/J-*NRP1*-HA-R, as described previously [73].

To create the *NRP1* HA tagged overexpression plasmid under MTT1 promoter, the complete coding sequence of *NRP1* was amplified using the OE*-NRP1*-F and OE-*NRP1*-R. The amplified sequences were digested with *BamH* I and *Asc* I and cloned into the pXS75 vector digested with the same enzymes. The constructs were introduced into CU428 and B2086 cells by biolistic transformation and mutants were screened under increasing paromomycin concentrations until cells failed to grow. The mutants were confirmed by PCR amplification using primers the OE-J-*NRP1*-F/OE-J-*NRP1*-R.

### Somatic knockout of *NRP1*

The 5′ flanking sequence and 3′ flanking sequence of *NRP1* were amplified from genomic DNA by PCR using the primers KO*-NRP1*-5′F/KO-*NRP1*-5′-R and KO*-NRP1*-3′-F/KO-*NRP1*-3′-R, respectively. The 5′ and 3′ flanking sequences were digested with *Sac* I/*Not* I and *Xho* I/*Kpn* I, respectively, and then ligated with the pNeo4 vector digested with the same enzymes. *NRP1* knockout strains were selected based on paromomycin resistance and PCR amplification using the primers J-KO-*NRP1*-F and J-KO-*NRP1*-R.

### Knockdown of *NRP1*

The *NRP1* knockdown construct was created by cloning the 490 bp region of the *NRP1* into the RNAi vector phpNeo. The forward and reverse fragments were amplified by primers RNAi-*NRP1*1F/RNAi-*NRP1*1R and primers RNAi-*NRP1*2F/RNAi-*NRP1*2R, respectively. The two fragments digested with the enzymes were cloned into the RNAi vector phpNeo digested with the same enzymes, respectively. The recombinant plasmid was digested using *Not* I and *Xho* I and introduced into the starved *Tetrahymena* cells. The transformed cells were selected under increasing paromomycin concentrations until cells failed to grow [43, 44]. *NRP1* knockdown efficiency was detected by qRT-PCR.

### RNA extraction and qRT-PCR

Total RNA was extracted from approximately 8 × 10^5^ cells with a Trizol reagent (Takara Biotechnology, Dalian, China). cDNA was synthesized with a random primer using the PrimerScriptTM RT reagent kit (Takara Biotechnology, Dalian, China). qRT-PCR was performed with the RT-*NRP1*-F/RT-*NRP1*-R primer pairs using an SYBR Green II mix (SYBR®Premix Ex Taq™ Kit, Takara). The steps are as follows: heat for 30 s at 95 °C, followed by 40 cycles of 5 s at 95 °C and an extension for 30 s at 60 °C. The relative quantifications of the *NRP1* mRNA expression levels were normalized using 17S rRNA as an internal control.

### Indirect immunofluorescence

For HA-Nrp1 localization, 5 mL of cell culture was collected (3500 rpm/4.5 min) and fixed in Lavdowsky’s fixative (ethanol:formalin:acetic acid:water, 50:10:1:39) overnight at 4 °C. For the alpha-tubulin or γ-H2A.X immunofluorescence staining, cells were fixed in 10 mM Tris pH 7.4 with 20 μL of Schaudinn’s fixative (2:1, saturated HgCl_2_:100% ethanol) overnight at 4 °C. The fixed cells (50 μL) were immobilized on cover glasses coated with poly-L-lysine (Sigma) and dried for 45 min at room temperature (RT). Coverslips were washed with phosphate-buffered saline (PBS), and fixed with 0.1% Tween-20 (PBST) for 10 min at RT. The samples were blocked in the blocking solution (3% BSA, 10% normal goat serum, 0.1% Tween-20 in PBS) for 1 h and then incubated with mouse anti-HA antibody (1:500, Millipore, USA), anti-α-tubulin mouse monoclonal primary antibody (1:200 dilution; T6074, Sigma, Santa Clara, USA), anti-γ-H2A.X mouse monoclonal antibody (1:200, Clone 2F3, BioLegend, USA) at 4 °C overnight. After washing three times with PBST, the samples were incubated with TRITC conjugated anti-mouse IgG antibody (1:800, Millipore, Billerica, MA, USA) for 1 h at RT. Coverslips were washed three times with PBST, then stained with 1 μg/mL DAPI for 15 min at RT. Digital images were collected using a Delta Vision Elite deconvolution microscope system (Applied Precision/GE Healthcare), confocal microscope (FV1000, OLYMPUS, Japan) or fluorescent microscope (BX51, OLYMPUS, Japan).

### Micronuclear integrity assay

The integrity of five micronuclear chromosomes was analyzed using 10 pairs of specific primers flanking chromosome breakage sequences by PCR [33]. The PCR cycling conditions were as follows: 5 min at 94 °C, 32 cycles of 30 s at 94 °C, 30 s at 56 °C, 1 min at 68 °C, and 5 min at 68 °C.

### Viability assay

The single cells were aspirated into the drops of SPP and incubated for 4 days at 30 °C. The drops were then transferred into individual wells of 96-well plates and cultured. The cells were observed through a stereo microscope (SZX16, OLYMPUS, Japan) after 4 days of culture at 30 °C.

### Co-immunoprecipitation

*Tetrahymena* was grown in 100 mL of 1 × SPP to a final concentration of 3.5 × 10^5^ cells/mL and was collected. Then, 24 h starved cells of different mating types were mixed in equal numbers to induce conjugation. After 2 h post-mixing, cells were collected, the pellets were re-suspended in 100 μl Tris-HCl (10 mM, pH 7.5) with 1 μL of 100 × Inhibitor Cocktail (Thermo) and 1 μL of 100 × 0.5 M EDTA, then frozen at − 80 °C. The pellets were thawed on ice and lysed by sonication. The whole cell lysate was clarified by centrifugation at 13,000 g for 20 min. The resulting soluble material was incubated with 20 μL of packed anti-HA agarose (Thermo) at 4 °C overnight. The anti-HA agarose was washed once with 3 mL of TBS plus 0.05% Tween-20. Then, 0.5 ml of TBS-T was added to each column and the columns were centrifuged for 10 s. The wash was repeated five times. The washes were saved for future analysis. Exactly 25 μL of 2 × non-reducing sample buffer was added to the anti-HA agarose. The spin column was heated at 100 °C for 5 min, and the sample was collected by centrifugation for 10 s. The sample was detected by SDS-PAGE and Western blot analysis. Another part of the sample was analyzed by MS. 5 μL product of protein digestion was used for LC-MS/MS analysis and separated using a nanoliter HPLC EASY-nLC1000 system (Thermo Fisher Scientific, MA, USA) couple with a Q Exactive mass spectrometer (Thermo Scientific, CA, USA). The mobile phase A was 0.1% formic acid solution with 2% acetonitrile, and the mobile phase B was 0.1% formic acid solution with 84% acetonitrile. The chromatographic column EASY column SC200 150 μm × 100 mm (RP-C18) was balanced with 100% A solution. The sample was loaded onto the EASY column SC001 traps 150 μm × 20 mm (RP-C18) through an automatic sampler and separated by a chromatographic column at the flow rate of 400 nL/min. The gradient elution procedure was as follows: the percentage change of the mobile phase B for 0–100, 100–108, and 108–120 min is 0–45%, 45–100%, and 100% of the linear change, respectively. After the capillary separation by HPLC, the product of protein digestion was determined using Q-Exactive mass spectrometer with the following parameters: duration: 120 min; detection method: positive ion detection; parent ion scanning scope: 300–1800 m/z; MS1 resolution at M/Z 200: 70,000; and MS2 resolution at M/Z 200: 17,500. The mass-to-charge ratios of polypeptides and polypeptide fragments were obtained by collecting 20 fragment patterns (MS2 scan, HCD) after each full scan. The data of MS were analyzed by Mascot2.2 Software. MS assay was supported by Shanghai Applied Protein Technology (Shanghai, China). All the proteins detected in the WT data sets were removed. Proteins in tables were selected either by a high number of hits or by a previously described functional or biochemical association with Nrp1 in other eukaryotes.

### Protein expression and pull down assay

The full-length *ASF1* and *NRP1* coding sequence was synthesized for expression in *Escherichia coli*. The synthesized *ASF1* and *NRP1* were cloned into pGEX-4T-1 and pESUMO, respectively. The recombinant plasmids were transformed into *Escherichia coli* BL21(DE3) and expressed under 0.1 mM IPTG induction at 37 °C or 16 °C. Recombinant proteins were purified by affinity chromatography [74]. Pull-down assays were performed in 200 µL buffer A200 (25 mM Tris-HCl, 10% glycerol, 0.01% Nonidet-P40, 1 mM Na_2_EDTA, 200 mM NaCl) containing 2 µg of His-Nrp1 and 1 µg of GST-Asf1. After overnight incubation at 4 °C, the beads were washed four times with 5 mL of A300 (25 mM Tris-HCl, 10% glycerol, 0.01% Nonidet-P40, 1 mM Na_2_EDTA, 300 mM NaCl). The binding protein was eluted and determined by 12.5% SDS-PAGE and Western blotting.

## Supplementary Information

**Additional file 1.** Additional figures S1–S8 and tables S1, S2.

## Data Availability

All relevant data are within the paper and its additional files. The data used to support the findings of this study are available upon reasonable request.
