# Supplementary material for: The histone chaperone Nrp1 is required for chromatin stability and nuclear division in Tetrahymena thermophila
Source: Epigenetics Chromatin. 2021 Jul 23;14:34. doi: 10.1186/s13072-021-00409-4 (PMC8299592; doi:10.1186/s13072-021-00409-4)
Supplement: Supplementary file 1 — Additional file 1. Additional figures S1–S8 and tables S1, S2. [file 13072_2021_409_MOESM1_ESM.docx]

**The Histone chaperone Nrp1 is required for chromatin stability and nuclear division in *Tetrahymena thermophila***

Yingjie Lian ^1^, Huijuan Hao^1^, Jing Xu^1, 2^, Tao Bo ^1^, Aihua Liang ^1^, Wei Wang ^1^*

Supplemental Information
Supplemental Figure Legends
Supplemental Figures S1-S8
Supplemental Tables S1-S2

**Supplemental Figure S1**

**Multiple sequence alignment of Nrp1 proteins from different model organisms.**

(A) Multiple sequence alignment of Nrp1s from (*T.t* (*Tetrahymena thermophila*), *P.t* (*Paramecium* *tetraurelia*), *A.t* (*Arabidopsis thaliana*), *C.e* (*Caenorhabditis elegans*), *D.m* (*Drosophila melanogaster*), *H.s* (*Homo sapiens*), *S.p* (*Schizosaccharomyces pombe*), *S.c* (*Saccharomyces cerevisiae*), *X.l* (*Xenopus lqevis*)). Residues are colored according to conservative.

(B) **Multiple sequence alignment of Nrp1 proteins from 7 different *Tetrahymena* species*.*** TPR domains are indicated. Residues are colored according to Clustalx multiple sequence alignment.

**Supplemental Figure S2**

**（A）Expression profile of the *NRP1*.** The expression profile microarray data are obtained from the *Tetrahymena* Functional Genomics Database (http://tfgd.ihb.ac.cn/). The y-axis indicates microarray expression values. The x-axis indicates developmental stages.

(B) **Heat map of transcript abundance of *NRP1* and histones in *Tetrahymena*.** The data are obtained from the *Tetrahymena* Functional Genomics Database (http://tfgd.ihb.ac.cn/). L1-L3: vegetative growth stage (~1×10^5^cells/ml, ~3.5 ×10^5^cells/ml and ~1×10^6^ cells/ml), S0-24: starvation stage (at 0, 3, 6, 9, 12, 15 and 24 h), and C: conjugation stage (at 0, 2, 4, 6, 8, 10, 12, 14, 16 and 18 h after mixing).

**Supplemental Figure S3**

**(A) HA antibody specific detection.** Indirect immunofluorescence of untagged cells during vegetative growth stage and pair formation stage using HA antibody. Scale bar, 10 µm.

**(B) Western blot analysis of Nrp1-HA.** Whole cell extracts made from WT and Nrp1-HA cells from vegetative growth stage using anti-HA antibody. α-Tubulin is a loading control on Western blot analysis.

**Supplemental Figure S4**

***NRP1* is essential for proliferation of *T. thermophila*.** (A) Schematic of the *NRP1* knockout. *NRP1* was replaced by Neo4 cassette by homologous recombination. (B) Identification of *NRP1* knockout mutants. Total DNA was isolated from wild-type CU428 and somatic *NRP1* knockout cells. KO-*NRP1*-B and KO-*NRP1*-C are different mating type mutants from B2086 and CU428 respectively. Different lanes represent different clones, M：molecular weight markers；lane1-3, KO-*NRP1*-B cells; lane4, wild type cells; lane5-9, KO-*NRP1*-C cells. The target gene was amplified by PCR. The arrow indicates recombination band and wild-type band. (C) *NRP1* knockout mutants recover into WT cells. Identification of *NRP1* knockout mutants under paromomycin-free medium culture. M：molecular weight markers；lane1-4, KO-*NRP1*-B cells; lane5, wild type cells; lane6-8, KO-*NRP1*-C cells.

**Supplemental Figure S5**

***NRP1* knockdown inhibits cellular survival and proliferation.** The single cell was aspirated into the drops of SPP and incubated for 4 days at 30 °C. The drops were then transferred into individual wells of 96-well plates and cultured. The proliferation of cells was observed through a stereo microscope. n =200.

**Supplemental Figure S6**

**Localization** **of γ-H2A.X during early meiotic stage.** Immunofluorescence staining of γ-H2A.X in WT cells and *nrp1*i cells. γ-H2A.X was used as an indicator of DNA double-strand breaks (DSBs). DNA was stained with DAPI. Scale bar, 10 µm.

**Supplemental Figure S7**

**Western blotting analysis of Nrp1-HA.** The whole cell extracts (WCE) of WT and Nrp1 mutant were prepared. Top panel was probed with anti-HA antibody, the bottom panel was probed with anti-tubulin antibody as a loading control.

**Supplemental Figure S8**

**Localization** **of HA-Nrp1 during** **late conjugation stage.** (A) Schematic diagram of OE-HA-*NRP1* construction. (B) OE-*NRP1*-B and OE-*NRP1*-C mutants were confirmed by PCR. Arrows indicate mutants (2200 bp) and WT (750 bp). M：molecular weight markers；lane1, lane3, wild type cells; lane2, lane4, OE-*NRP1*-B cells; lane5-8, OE-*NRP1*-C cells. (C) Immunofluorescence staining of overexpressing HA-Nrp1 mutants. HA-Nrp1 was overexpressed under MTT1 promoter with Cd^2+^ induction. DNA was stained with DAPI. Arrowheads indicate old MACs, stars indicate anlagen. Scale bar, 10 µm.

Supplementary Figure 1

A


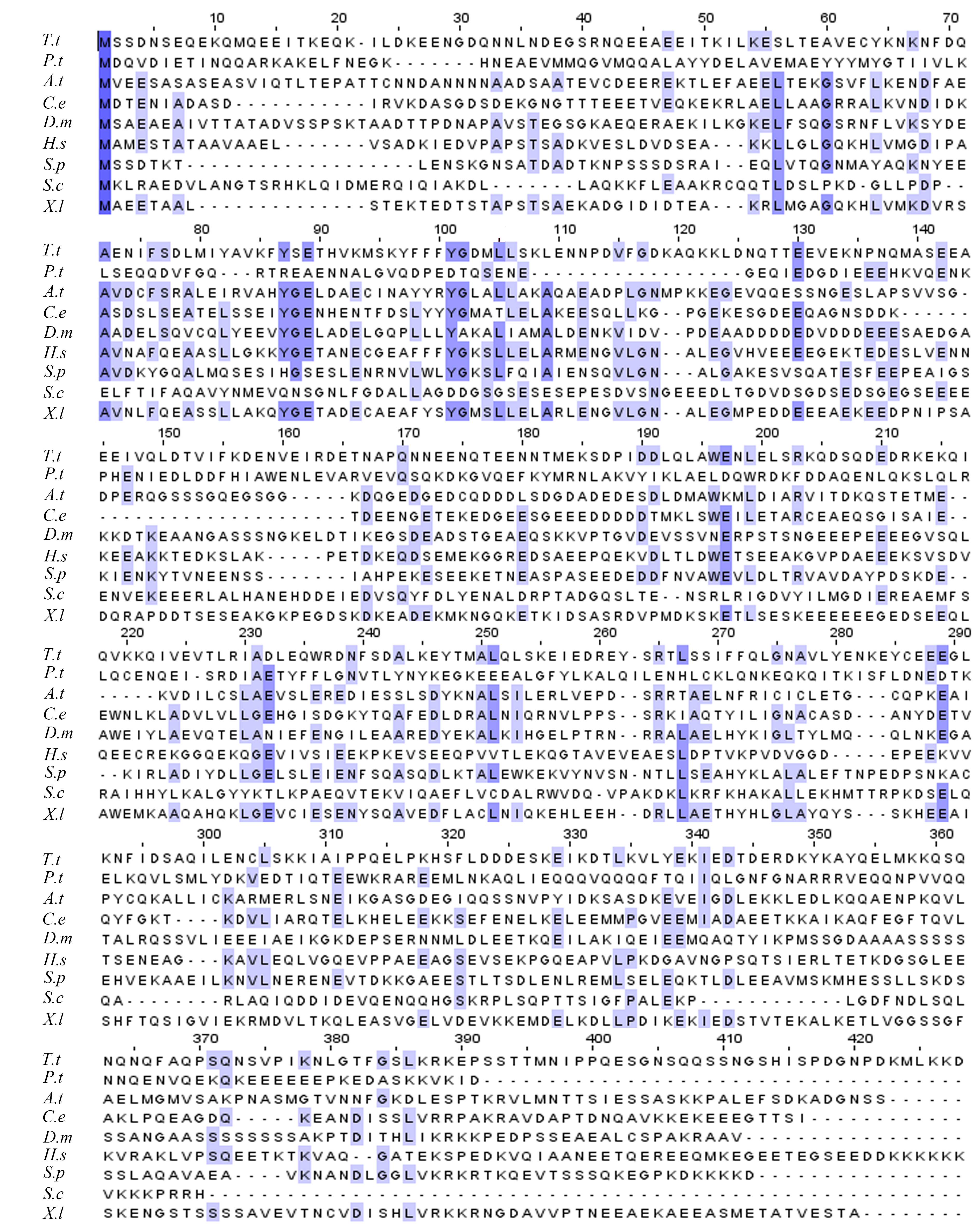


**B**


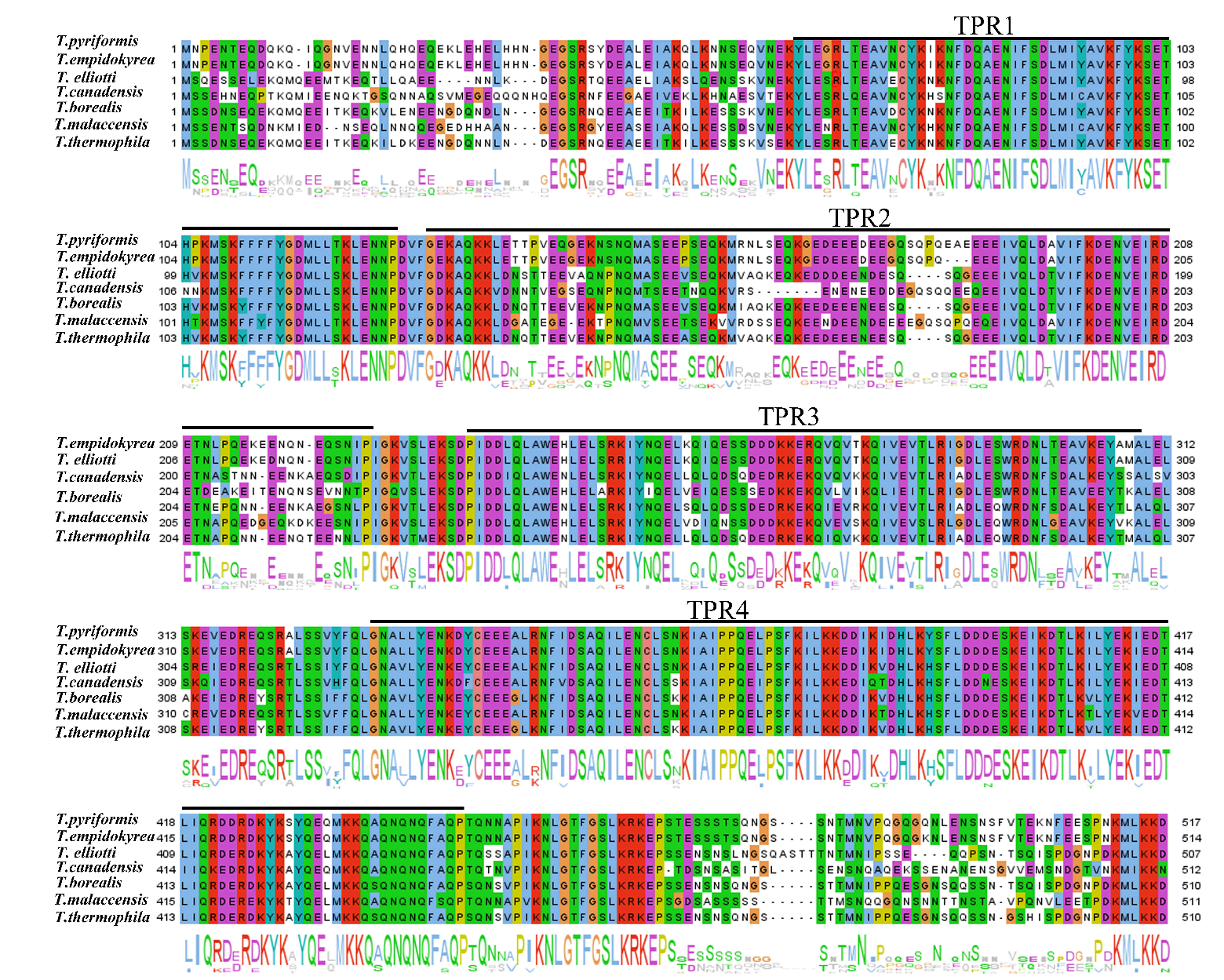


Supplementary Figure 2


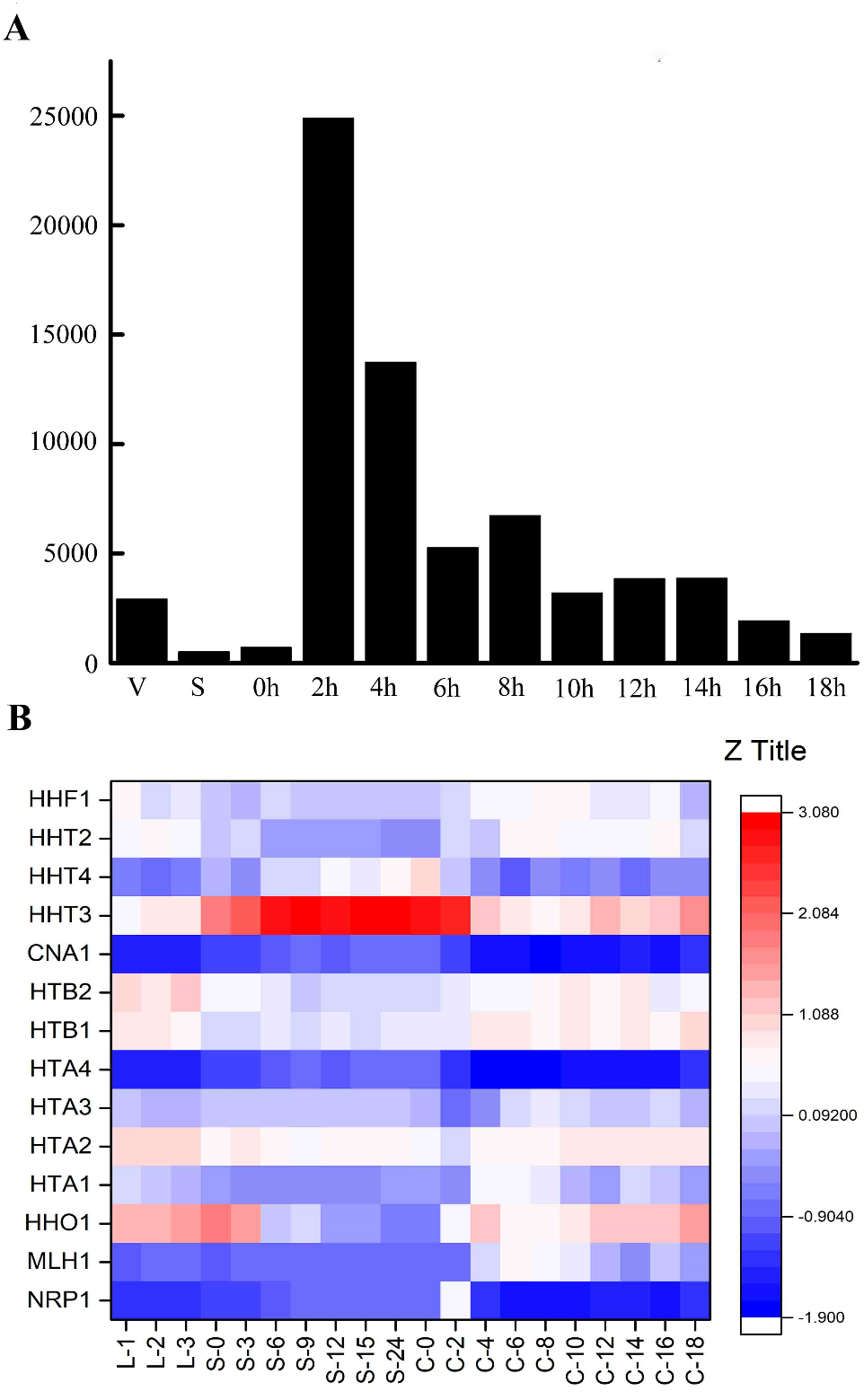


Supplementary Figure 3


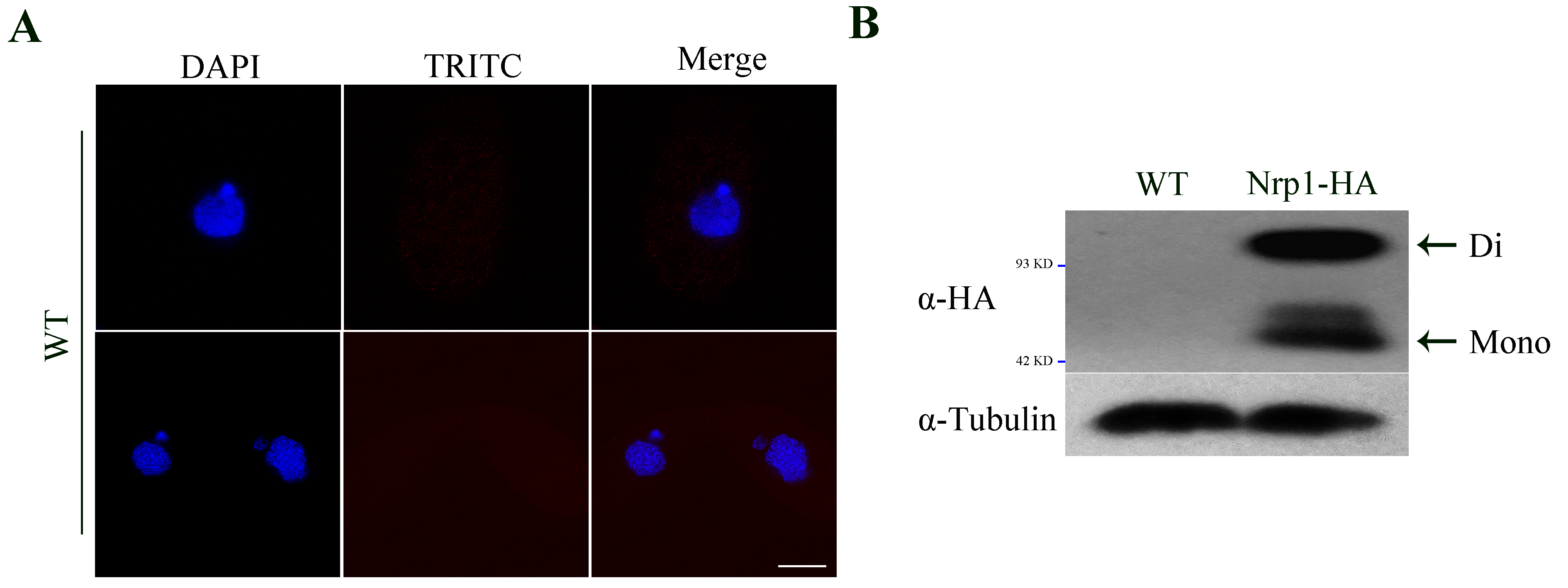


Supplementary Figure 4


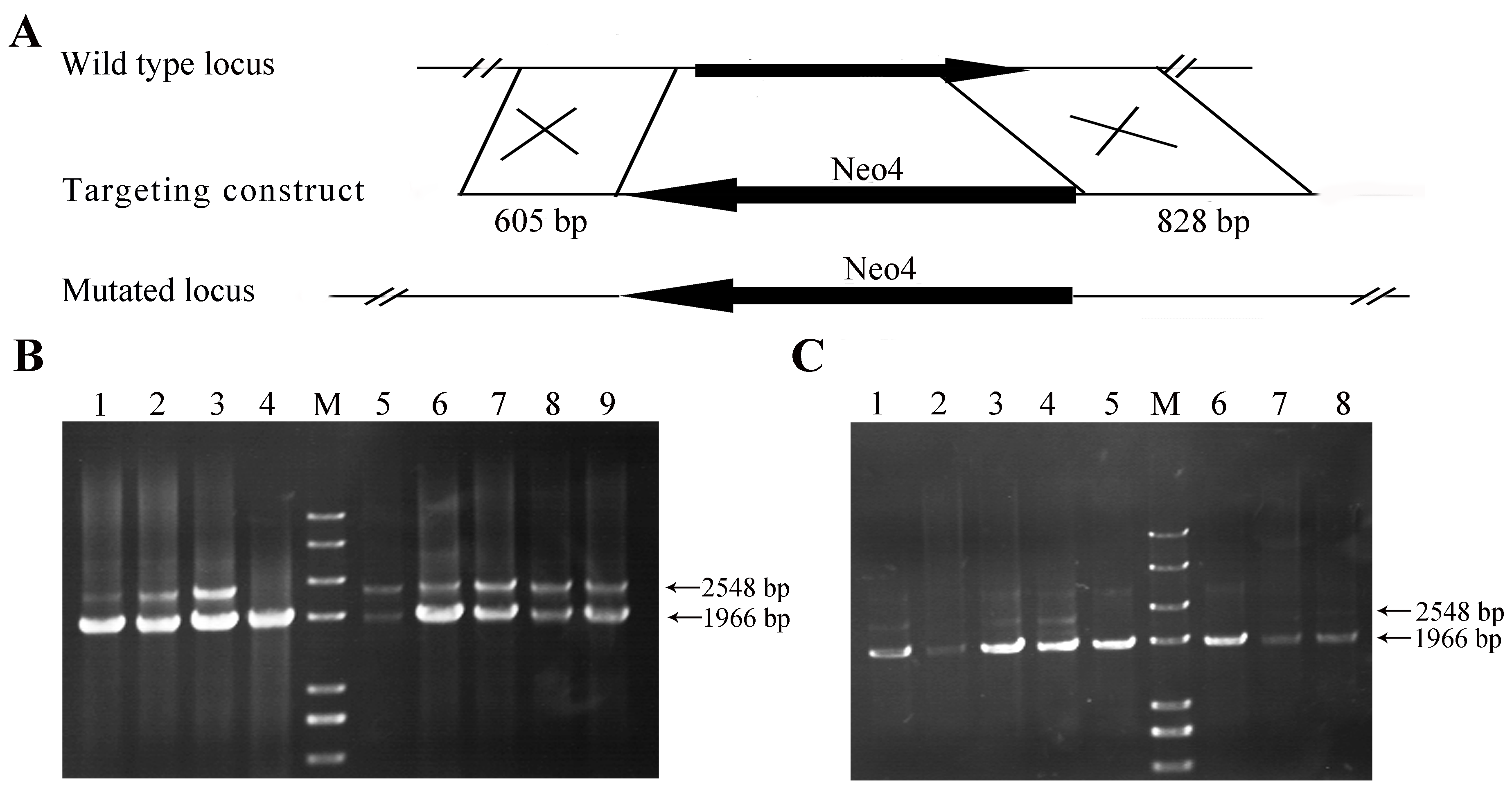


Supplementary Figure 5


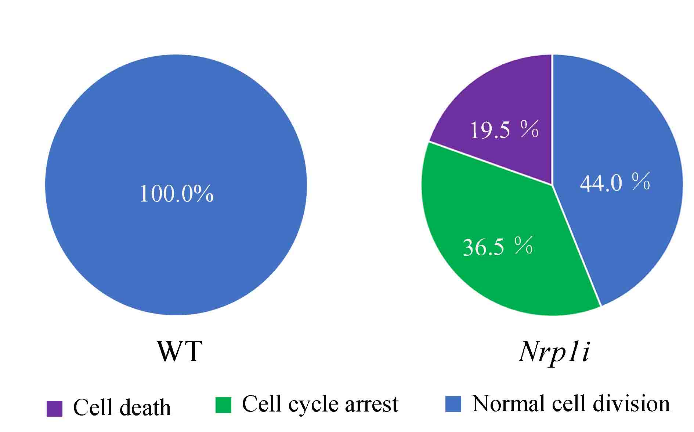


Supplementary Figure 6


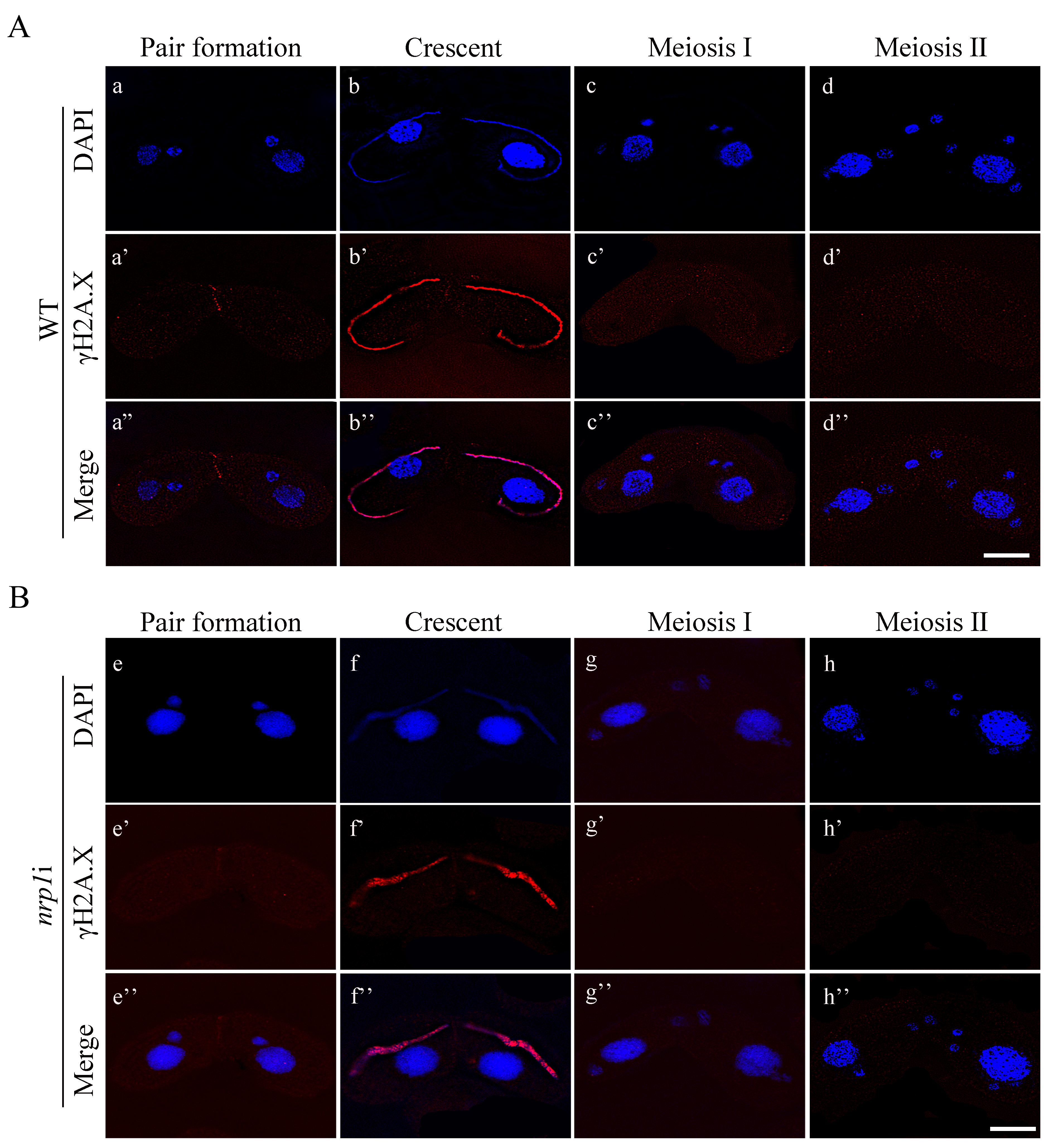


Supplementary Figure 7


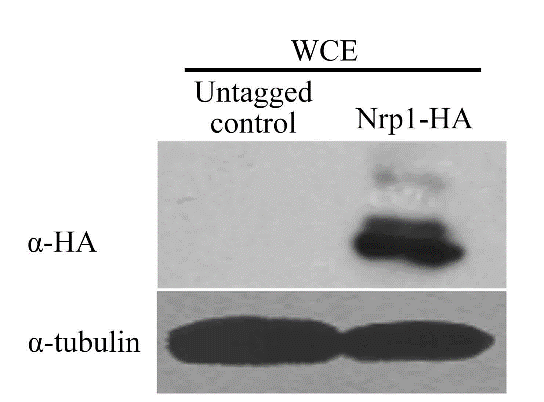


Supplementary Figure 8


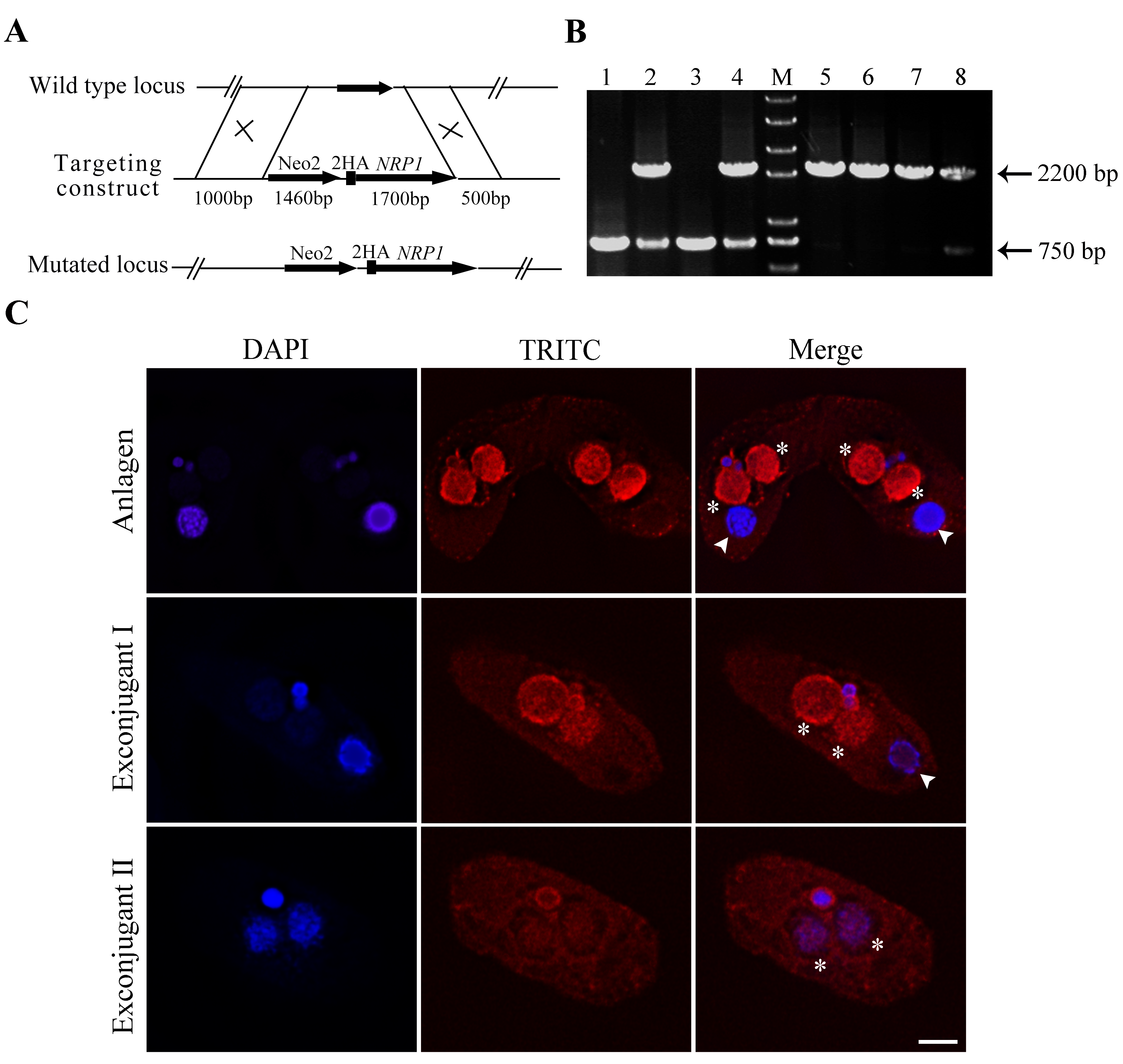


Supplementary Table S1: Primers used in this work

| PCR primers | Sequence |
| --- | --- |
| *NRP1-HA*-F1 | GAGCTCATAAGATAGCTAAGATGAGGATAG |
| *NRP1-HA*-R1 | GCGGCCGCATCCTTTTTGAGCATTTTATCTGG |
| *NRP1-HA*-F2 | CTCGAGGTAATAGCTTGCAACATCACTTTC |
| *NRP1-HA*-R2 | GGTACCTCACCATTTAAGCCTCCAGTAAC |
| *HA-NRP1*-F | GTCGAAGTAACTTTAAGAATTGCTG |
| *HA-NRP1*-R | ACTCTATGGTAGTTAGTCAACTATG |
| J-*NRP1-HA*-F | CCAGATAAAATGCTCAAAAAGGATT |
| J-*NRP1-HA*-R | GAAAGTGATGTTGCAAGCTATTAC |
| RNAi- *NRP1*1F | GTTTAAAC AGAATATTGTGAAGAAGAGGGTCTT |
| RNAi- *NRP1*1R | GGATCCCCTGATTCTTAAGGTGGGATATTC |
| RNAi- *NRP1* 2F | CCCGGGAGAATATTGTGAAGAAGAGGGTCTT |
| RNAi- *NRP1*2R | CTGCAGCCTGATTCTTAAGGTGGGATATTC |
| KO-*NRP1*-5’-F | GAGCTCAGCTCTTAGATAGATTTTATTTTT |
| KO-*NRP1*-5’-R | GCGGCCGCTTCAATAAATAAGATAAACTATAAC |
| KO-*NRP1*-3’-F | CTCGAGCAATTCCTCCTTAAGAACTTCCTAG |
| KO-*NRP1*-3’-R | GGTACCAAGATATCAAAATTAATCACTGAAGATC |
| J-KO-*NRP1*-F | ATTCTAATAGTTTCTGTGTAGCGG |
| J-KO-*NRP1*-R | TTAGGTAAGTTAATTAATCCTCAATATG |
| RT-*NRP1*-F | ACAAAGCTTACTAAGAACTTATGA |
| RT-*NRP1*-R | CATTAGATGATTGTTGACTATTACC |
| OE-*NRP1*-F | GGATCCATGAGTTCTGACAATAGTGAATAAG |
| OE-*NRP1*-R | GGCGCGCCTCAATCCTTTTTGAGCATTTTATCTG |
| OE-J-*NRP1*-F | GCTACGTGATTCACGATTTATGCAATG |
| OE-J-*NRP1*-R | CGAAACTGATTTTATGCAATTATGAATTAC |

Underline indicates recognition sites of the restriction enzymes.

Supplementary Table S2: Identification of the interactive proteins of Nrp1-HA by affinity purification combined with mass spectrometry analysis

| Gene ID | Prey | UniquePepCout |
| --- | --- | --- |
| TTHERM_00196370 | chaperonin CPN60_1 (Hsp60) | 7 |
| TTHERM_00444670 | heat shock protein HSP90 | 7 |
| TTHERM_000570549 | histone H3.1 | 3 |
| TTHERM_00189180 | Histone H3.2 | 3 |
| TTHERM_00016200 | histone H3.4 | 2 |
| TTHERM_00016170 | histone H3.3 | 2 |
| TTHERM_00442300 | histone chaperone asf1 | 2 |
| TTHERM_00786930 | nucleosome assembly protein | 4 |
| TTHERM_00283330 | metallopeptidase family M24 containing protein (CET1 SPT16) | 1 |
| TTHERM_00688660 | Histone binding protein RBBP4 or subunit C of Caf1 complex protein | 1 |
| TTHERM_00046920 | TBP interacting DNA helicase (RVB2) | 3 |
| TTHERM_00448570 | DNA replication licensing factor MCM6 | 1 |
| TTHERM_01207610 | MCM2 3 5 family protein | 1 |
| TTHERM_00865240 | DNA ligase I, ATP dependent protein (Lig1) | 1 |
| TTHERM_00142330 | DNA repair RAD51_like protein | 1 |
| TTHERM_00825440 | DNA topoisomerase IV (Top2) | 1 |
| TTHERM_00149190 | Ubiquitin activating enzyme E1 UAB14 | 3 |
| TTHERM_01341620 | Ubiquitin conjugating enzyme E2 (UBC4) | 1 |
| TTHERM_01123950 | Ubiquitin conjugating enzyme E2 (UCN2) | 1 |
| TTHERM_00647540 | acyltransferase family protein | 1 |
| TTHERM_00637650 | ELP3 family histone acetyltransferase | 1 |
| TTHERM_00388200 | spindle assembly abnormal like protein, putative (SAS6A) | 2 |
